# Zeolite-like liquid crystals

**DOI:** 10.1038/ncomms9637

**Published:** 2015-10-21

**Authors:** Silvio Poppe, Anne Lehmann, Alexander Scholte, Marko Prehm, Xiangbing Zeng, Goran Ungar, Carsten Tschierske

**Affiliations:** 1 Institute of Chemistry, Organic Chemistry, Martin Luther University Halle-Wittenberg, Kurt-Mothes Strasse 2, D-06120 Halle/Saale, Germany; 2 Department of Materials Science and Engineering, University of Sheffield, Mappin Street, Sheffield S1 3JD, UK; 3 Department of Physics, Zhejiang Sci-Tech University, Xiasha College Park, 310018 Hangzhou, China

## Abstract

Zeolites represent inorganic solid-state materials with porous structures of fascinating complexity. Recently, significant progress was made by reticular synthesis of related organic solid-state materials, such as metal-organic or covalent organic frameworks. Herein we go a step further and report the first example of a fluid honeycomb mimicking a zeolitic framework. In this unique self-assembled liquid crystalline structure, transverse-lying π-conjugated rod-like molecules form pentagonal channels, encircling larger octagonal channels, a structural motif also found in some zeolites. Additional bundles of coaxial molecules penetrate the centres of the larger channels, unreachable by chains attached to the honeycomb framework. This creates a unique fluid hybrid structure combining positive and negative anisotropies, providing the potential for tuning the directionality of anisotropic optical, electrical and magnetic properties. This work also demonstrates a new approach to complex soft-matter self-assembly, by using frustration between space filling and the entropic penalty of chain extension.

## Abstract (web summary)

Zeolites with regular porous structures are widely used as gas adsorbents and scaffolding for catalysts. Poppe *et al*. report a liquid crystal with zeolite-like structure by self-assembly of polyphilic molecules with π-conjugated rod-like cores into a honeycomb formed by pentagonal/octagonal channels.

## Graphical abstract

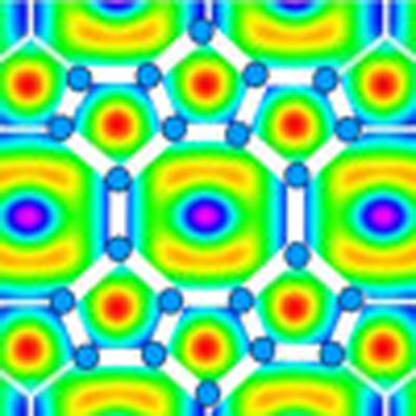

Zeolites are well-known inorganic solid-state materials with well-defined porous framework structures, used for gas storage, separation and catalytic applications^1^,^2^. For control of micro porosity, metal-organic frameworks (MOFs)^3^ and covalent organic frameworks (COFs) were developed in the previous decade^4^,^5^. In addition, COFs and MOFs recently received significant attention for optoelectronic and photovoltaic applications^5^,^6^,^7^ and as ferroelectrics^8^, in these cases not necessarily requiring open pores. For the latter applications, the framework structures provide the facility to tailor the spatial organization of π-conjugated aromatic groups in a desired pattern. Related soft structures, representing liquid crystal (LC) honeycombs, have previously been developed for T-shaped and X-shaped polyphilic molecules (see Fig. 1a,b)^9^,^10^,^11^. In these self-assembled soft structures, rigid aromatic rod-like cores of the molecules forming the honeycomb walls are connected by dynamic intermolecular hydrogen bonding between their glycerol end groups. The honeycomb channels are filled with flexible chains laterally attached to the aromatic rods. As shown in previous work, the volume of the flexible chains relative to the rod length determines the size and shape of the channel cross-section, which range from triangular to hexagonal and beyond^9^,^10^,^11^. The cross-sectional shape of the channels is mainly determined by geometric rules, whereas the hydrogen bonding, the segregation of polar from apolar units and rigid from flexible segments, as well as anisotropic interactions between the rod-like cores, contribute to the stabilization of the periodic honeycomb structures. Putting it crudely, although the periodic tiling in the *xy* plane ensures low energy, the disorder along *z* axis secures relatively high entropy. This leads to thermodynamically stable bulk structures with sufficient mobility to allow efficient self-healing of local defects. They are also sufficiently robust to be independent of most molecular or atomistic features of the substrate surface, in contrast to related two-dimensional (2D) assemblies of molecules^12^,^13^, nanoparticles or colloidal particles on solid or LC surfaces^14^,^15^.

Formation of periodic structures combining channels of different shapes was previously observed for related polyphilic X-shaped molecules (Fig. 1a) with two different and strongly incompatible side chains, such as perfluoroalkyls and carbosilanes, attached to opposite sides of the rod-like core^16^,^17^,^18^. As these chains cannot mix, superstructures combining channels with different size and content were obtained by combining incompatible chains with widely disparate volumes. For T-shaped molecules (Fig. 1b) with only one chain or X-shaped molecules with two identical chains, mainly congruent honeycombs, consisting of identical channels, were obtained. The only periodic mixed-cell honeycombs observed in these compounds were triangle–square combinations (Fig. 1b) in cases where the chains were too large for triangular, but too small for square channels^19^.

Here we present a new approach of deliberately introducing packing frustration by keeping the volume but shortening the length of the flexible chains. Not only do we get soft honeycombs with largely disparate cells (pentagonal and octagonal), but the imposed frustration also results in an unprecedented type of LC where mesogens orient both perpendicular and parallel to the director axis. This opens the possibilities for a range of fascinating and surprising new complex structures through self-assembly of T-shaped molecules with only a single branched chain (structure **c** in Fig. 1). One of them represents the first example of a zeolite-like nanostructured LC that forms by self-assembly of polyphilic molecules involving π-conjugated rod-like cores and combining pentagonal with octagonal channels. Another novel structure is also reported, combining pentagonal and hexagonal channels. These complex superstructures are the result of a newly adopted strategy by introducing entropy controlled packing frustration to the previously developed concept of LC honeycombs.

## Results

### Molecular structures and LC phases

**Figure 2:**
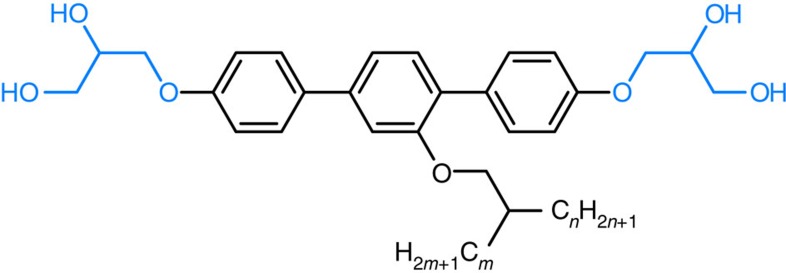
Molecular structure of compounds *m*/*n*. All compounds have the same number of carbon atoms in the branched lateral chain with *m*+*n*=20; the data are collated in Table 1.

Five compounds with structure **c**, designated by the length of the two branches in their lateral chains as ***m/n*** (see Fig. 2 and Table 1), were synthesized as described in the Methods and the Supplementary Methods; the differential scanning calorimetry (DSC) traces are shown in Supplementary Fig. 1. All compounds are isomers with *m*+*n*=20 but differing in the length of the two branches (*m*, *n*); they range from compound **20/0** having a linear *n*-alkyl chain to compound **10/10** having two equal branches. As shown in Table 1, all compounds form enantiotropic (thermodynamically stable) liquid crystalline phases, in most cases over relatively wide temperature ranges between the crystalline (Cr) and the isotropic liquid state. Regarding X-ray diffraction, these ordered fluids, as expected, show only diffuse wide-angle scattering with a maximum at *d*=0.45–0.46 nm. This corresponds to the mean lateral distance between the molecules and indicates that the molecules have no fixed position. The presence of sharp Bragg reflections in the small angle range however confirm the presence of long-range 2D mesoscopic order. The plane group assignments of the LC phases of compounds ***m/n*** are shown in Table 1.

### Hexagonal honeycombs

**Figure 3:**
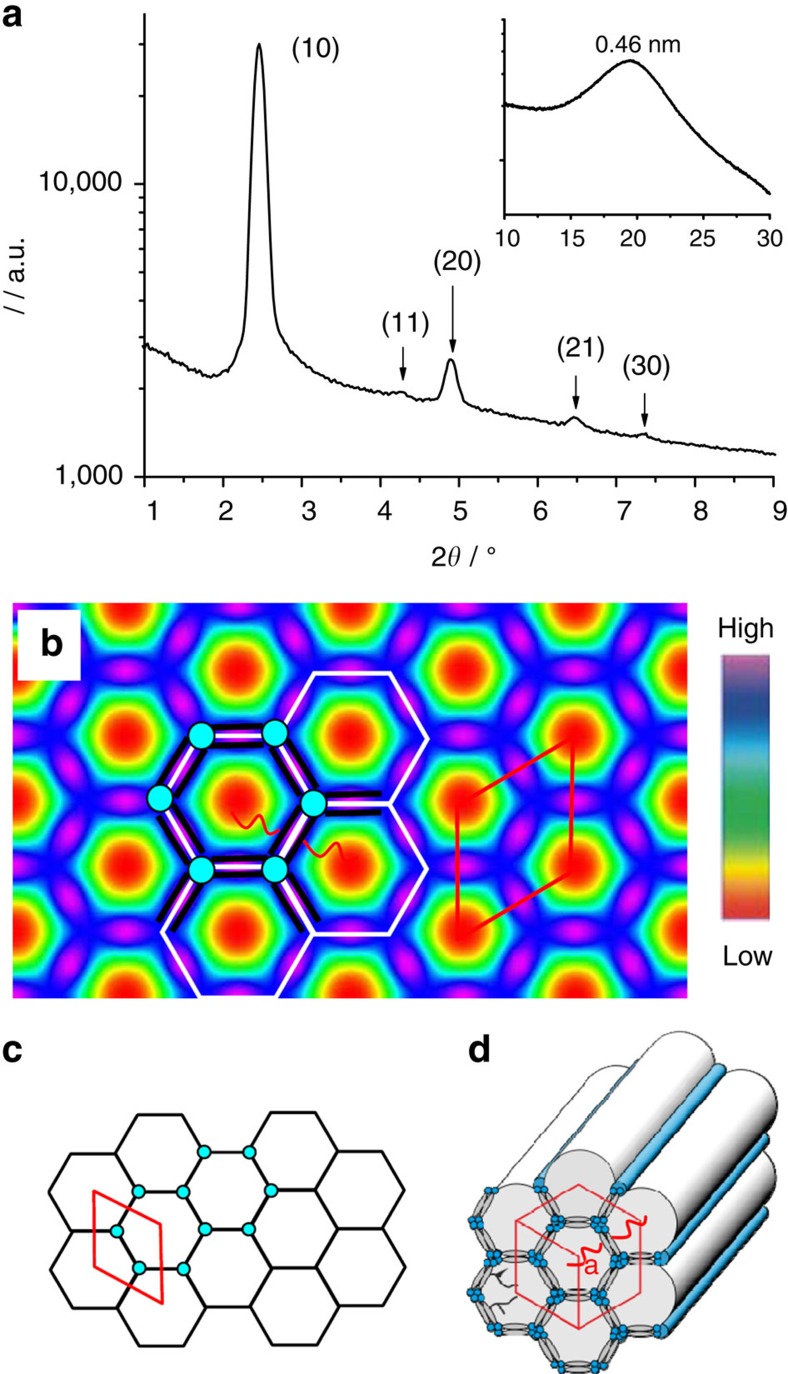
Hexagonal honeycomb. (**a**) 2*θ* scan of the small-angle X-ray diffraction (XRD) pattern of the Col_hex_^1^ phase of **16/4** at *T*=135 °C (inset shows wide angle region), (**b**) the corresponding electron density map, (**c**) hexagonal net and (**d**) a three-dimensional model showing molecular organization in the hexagonal honeycomb; for additional XRD data of **16/4**, see Supplementary Fig. 5 and Supplementary Table 3, and alternative electron density maps based on other phase combinations are shown in the Supplementary Fig. 8; for XRD data of compound **20/0** with the same Col_hex_^1^ structure, see Supplementary Fig. 4; for estimation of the number of molecules in the unit cell and honeycomb walls of the Col_hex_^1^ phases, see Supplementary Note 4 and Supplementary Table 6.

For compounds **20/0** with a linear chain and **16/4** having the branching point closest to one end of the alkyl chain, the 1/*d* values of the small angle Bragg reflections are in the ratio 1:
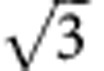
:2:
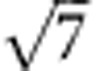
:3, in line with a hexagonal lattice with *a*_hex_=4.2 nm (Fig. 3a). In both cases, these correspond to *a*_hex_=
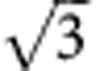
*L*_mol_, with *L*_mol_ being the length of the rod-like core unit between the ends of the glycerol groups in the most extended conformation (*L*_mol_=2.45 nm). This *d/L*_mol_ ratio is indicative of a regular hexagonal honeycomb involving six molecules in the circumference of each hexagonal channel (Col_hex_^1^, Fig. 3c,d)^9^,^10^. The reconstructed electron density map (Fig. 3b) confirms this structure and shows a high electron density net (purple) representing the hexagonally arranged aromatic-glycerol walls surrounding the low-electron-density cells (red/yellow) containing the lateral alkyl chains. The fact that the molecules are aligned transverse to the channels is confirmed by the negative birefringence (see Supplementary Fig. 2a). As typical for LC honeycombs of molecules with only one lateral group, the walls are formed by back-to-back organized pairs of molecules (Fig. 3b,d, see also Supplementary Table 6 and Supplementary Note 4).

### Hexagonal superlattice of compound **14/6**

**Figure 4:**
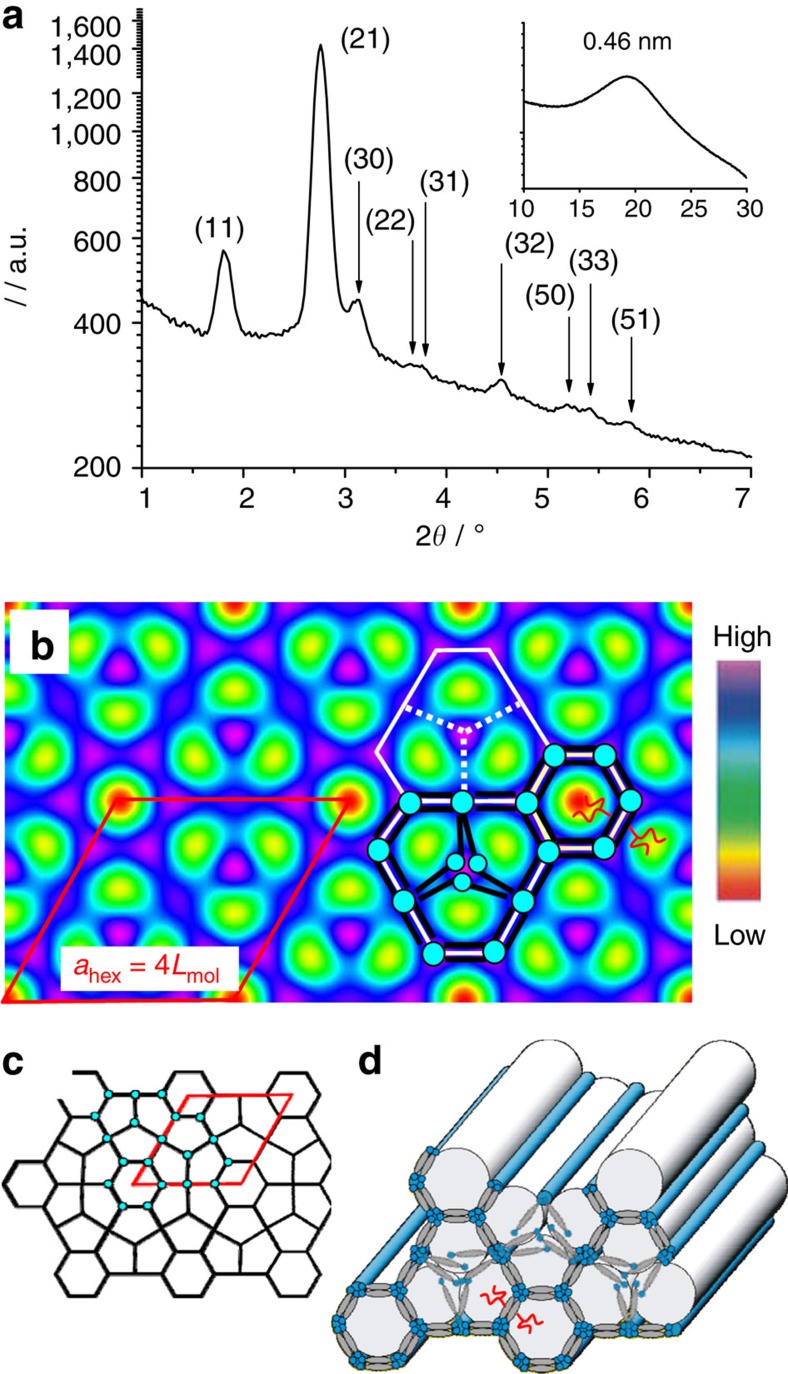
Hexagonal superlattice. (**a**) 2*θ* Scan of the X-ray diffraction (XRD) pattern of the Col_hex_^2^ structure of **14/6** at *T*=135 °C, (**b**) the corresponding electron density map, (**c**) the net-representation and (**d**) a three-dimensional model showing the molecular organization in the hexagonal superlattice formed by hexagonal and distorted pentagonal channels; see Supplementary Table 4 for tabular XRD data; Supplementary Fig. 9 shows alternative electron density maps based on other phase combinations and the phase choice is explained in Supplementary Note 1; Supplementary Table 6 gives the estimation of the number of molecules in the unit cell and honeycomb walls, which are explained in more detail in the Supplementary Notes 3 and 4.

For compound **14/6**, with a reduced difference between branches *m* and *n*, the small angle reflections between ∼130 and 145 °C can be indexed as (11), (21), (30), (22), (31), (32), (50), (33) and (51) of a hexagonal lattice. Interestingly, the (10) and (20) reflections are very weak or completely missing (Fig. 4a), which indicates a structure significantly different from that of Col_hex_^1^. Here, the hexagonal lattice parameter *a*_hex_=9.72 nm corresponds to about 4*L*_mol_; therefore, a simple hexagonal honeycomb as in **20/0** and **16/4** can be excluded. The electron density map (Fig. 4b) of this Col_hex_^2^ structure indicates the presence of a more complex superlattice. There are small regular hexagons with low-density alkyl centres (red), each surrounded by six giant hexagons with threefold symmetry and alternating side lengths, corresponding to *L*_mol_ and 2*L*_mol_, respectively (Fig. 4b–d). These latter hexagons, containing nine molecules in their circumference (9-hexagons, indicated by solid white lines in Fig. 4b), are further divided into triplets of pentagons (dotted lines). However, the pentagonal cells within the 9-hexagons appear to be poorly ordered, as the electron-density difference between their walls and their cell interior is reduced (green/blue). This suggests some structural disorder, which is likely to result from the extended (1.17-fold) length of those inner walls compared with the outer walls of the 9-hexagon (see Supplementary Note 3). This mismatch and the overcrowding of the pentagonal channels by the alkyl chains may be responsible for the disorder inside the 9-hexagons, which could provide an entropic advantage for this structure (see Fig. 4b,d).

### Zeolite-like LC with *c*2*mm*-lattice

**Figure 5:**
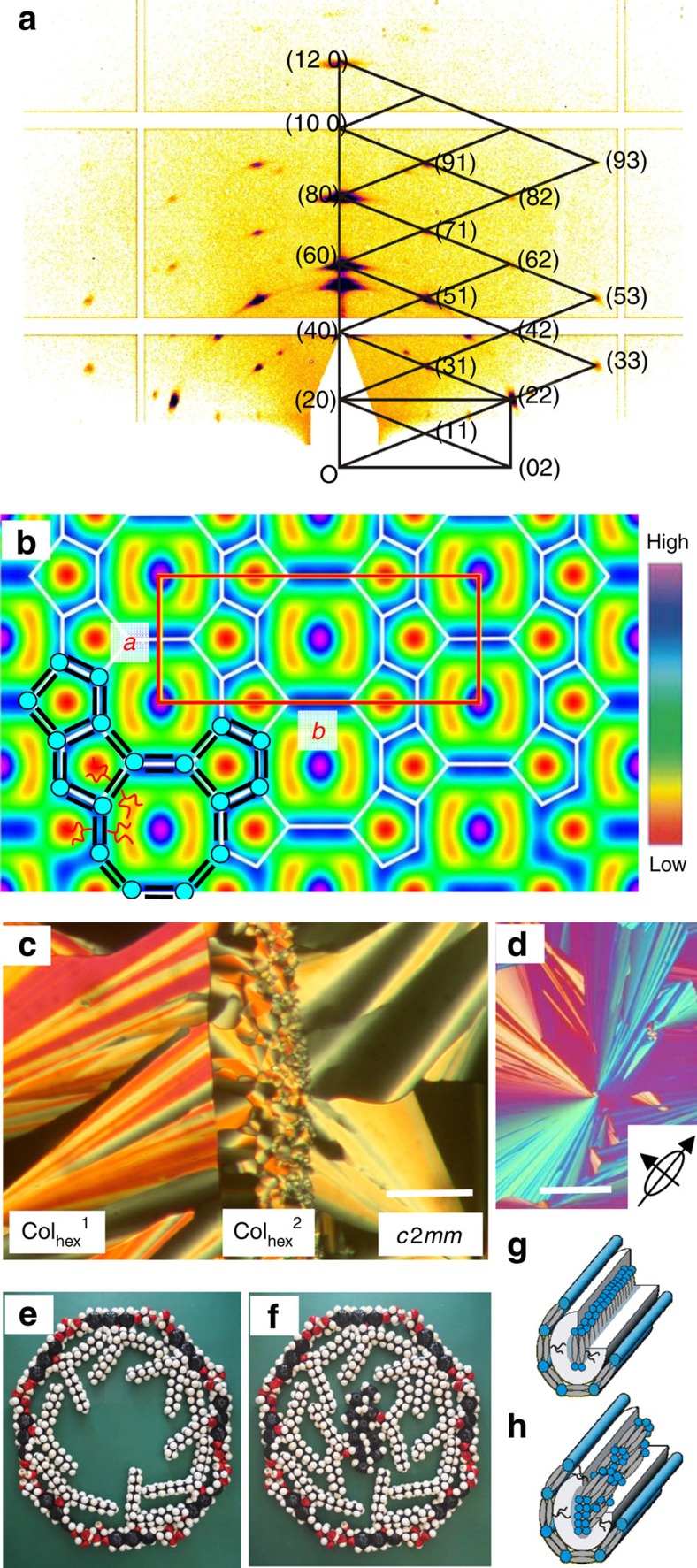
Zeolite-like organization of compounds **12/8** and **10/10** with *c*2*mm* lattice. (**a**) GISAXS pattern of **10/10** at *T*=90 °C with indices; (**b**) electron density map of **10/10** with colour scale, tiling pattern (white lines) and molecular arrangement (black lines and blue dots) are superimposed; (**c**) contact region between the low-birefringence *c*2*mm* phase of **12/8** (left) and the high-birefringence Col_hex_^1^ phase of compound **16/4** (right) with induced Col_hex_^2^ phase between them at *T*=136 °C (see also Supplementary Fig. 3); (**d**) texture as observed for **12/8** at 140 °C between crossed polarizers with *λ*-retarder plate; the indicatrix orientation is shown in the inset (see also Supplementary Fig. 2c,d and explanations in the corresponding caption); (**e**) molecular model showing eight molecules of **10/10** forming the walls of the octagonal cells; (**f**) the empty space being filled by ten additional coaxial molecules; (**g**,**h**) two possible orientations of molecules filling an octagonal channel with (**g**) a ribbon of transverse aligned molecules and (**h**) a co-axial bundle of rods; see Supplementary Tables 2 and 5 for tabular X-ray diffraction (XRD) data and Supplementary Figs 6 and 7 for additional diffraction patterns; Supplementary Fig. 10 shows alternative electron density maps based on other phase combinations and the phase choice is explained in Supplementary Note 2; Supplementary Table 6 gives the estimation of the number of molecules in the unit cell and honeycomb walls, which are explained in more detail in the Supplementary Note 4.

**Figure 6:**
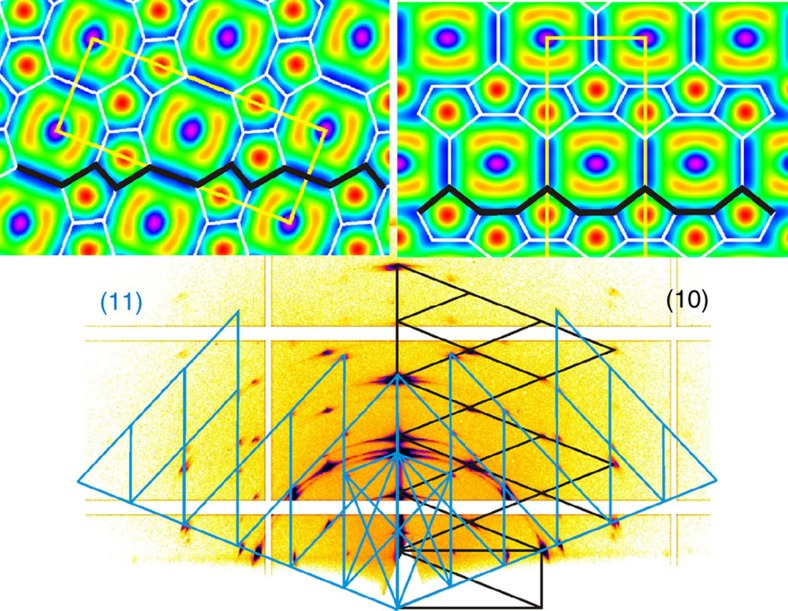
Different orientations of the *c*2*mm* latitice of **10/10**. GISAXS pattern of the Col_rec_/*c*2*mm* phase of **10/10** at *T*=90 °C, showing the two orientations (10) and (11) together with electron density maps oriented to lie on a {100} and {110} plane, respectively; the thick black lines show the contact plane.

The soft, self-assembled superstructure formed by compounds **10/10** and **12/8** having two branches with equal or similar length is very different from those of all other compounds. The small-angle X-ray scattering (SAXS) patterns show numerous Bragg reflections, which can be indexed on a rectangular *c*2*mm* lattice (conditions *hk*: *h*+*k*=2*n*: *h*0: *h*=2*n* and *k*0: *k*=2*n*, see Fig. 5a and Supplementary Figs. 6 and 7). The 2D electron density map of **10/10** (Fig. 5b) shows high-density dots (purple/blue) located in the centre and at the corners of the unit cell. Each is enclosed between two low-density crescents (red/yellow). Within the medium density continuum (green) there are additional low-density dots (red) arranged along wavy lines. These are partly surrounded by lines of highest density (blue), assigned to the glycerol groups and terphenyl cores. Superimposed on the map in Fig. 5b is the proposed arrangements of honeycomb walls (white), as are a few schematic molecules (black lines and blue dots). Accordingly, antiparallel pentagonal channels fuse to form layers. Each pentagonal channel is filled with low-density alkyl chains. The space between these layers is divided by additional walls into octagonal cells. With planar alignment (channels parallel to the substrate), two orientations are observed in the grazing incidence SAXS (GISAXS) patterns, having either the {100} or the {110} planes horizontal (see Fig. 6). This allows maximum contact between the polar groups and the horizontal polar substrate, with minimum lattice distortion^20^, thus further supporting the proposed nanostructure.

The high electron density in the centres of the octagonal cells indicates that the space in these giant channels contains additional molecules with the aromatic cores and glycerol units in the centre. In principle, there are two possible alignments of the additional molecules, either perpendicular or parallel to the channels (Fig. 5g, h). In the first case, quasi-infinite ribbons of transverse-oriented pairs of molecules arranged side-by-side run along the channels (Fig. 5g). In the second case, bundles of axially oriented molecules, about 7–11 in cross-section, stack end-to-end along the columns (see Fig. 5h, Supplementary Table 6 and explanations in Supplementary Note 4).

To distinguish these two cases, optical investigations were performed between crossed polarizers. All honeycombs show birefringent fan-like ‘developable domains' as typical for fluid superstructures with a 2D lattice^21^. In the fans, the channels are parallel or nearly parallel to the substrate and circle around the central defect. Figure 5c shows the textures of all three LC structures in a sample with constant thickness, the hexagonal honeycomb of **16/4** (Col_hex_^1^) on the left and the zeolitic *c*2*mm* structure of **12/8** on the right. In the contact region in the middle, where both compounds mix, the pentagon/hexagon tiling of the intermediate Col_hex_^2^ phase develops, the same phase as observed in compound **14/6**. The film thickness is such that in all areas first-order birefringence colours are seen. There is a distinct shift in colour of the fans from red/orange to yellow as one moves from the Col_hex_^1^ and Col_hex_^2^ to the *c*2*mm*. This implies reduced retardation, hence reduced birefringence of the zeolite-like *c*2*mm* structure, meaning that the positive contribution of the terphenyls in the centre of the octagonal channels is partially cancelling the negative contribution from the terphenyls in the honeycomb walls. Had all terphenyls been perpendicular to the channel axis, the absolute value of birefringence would not have been reduced.

Using an additional *λ*-retarder plate the direction of the intramolecular π-conjugation pathway with respect to the cylinder axis can be identified. As shown in Fig. 5d and Supplementary Fig. 2 the orientation of the blue-shifted fans is south-west to north-east, where the columns, being normal to the fans, lie southeast to northwest. The blue colour means that the slow axis is oriented south-west to north-east for all compounds ***m/n***, that is, it is perpendicular to the axis of the cylinders. Hence, the birefringence is negative, with the average orientation of the terphenyl cores perpendicular to the channels, in line with the proposed honeycomb structures. For some rays in the fans of the *c*2*mm* phase, the birefringence is virtually zero (red), even at 45° orientation to the polarizers (Fig. 5d), indicating that in this projection the contributions to birefringence of the transverse and axial molecules cancel almost exactly (see also Supplementary Fig. 2 and caption for more details).

## Discussion

**Figure 7:**
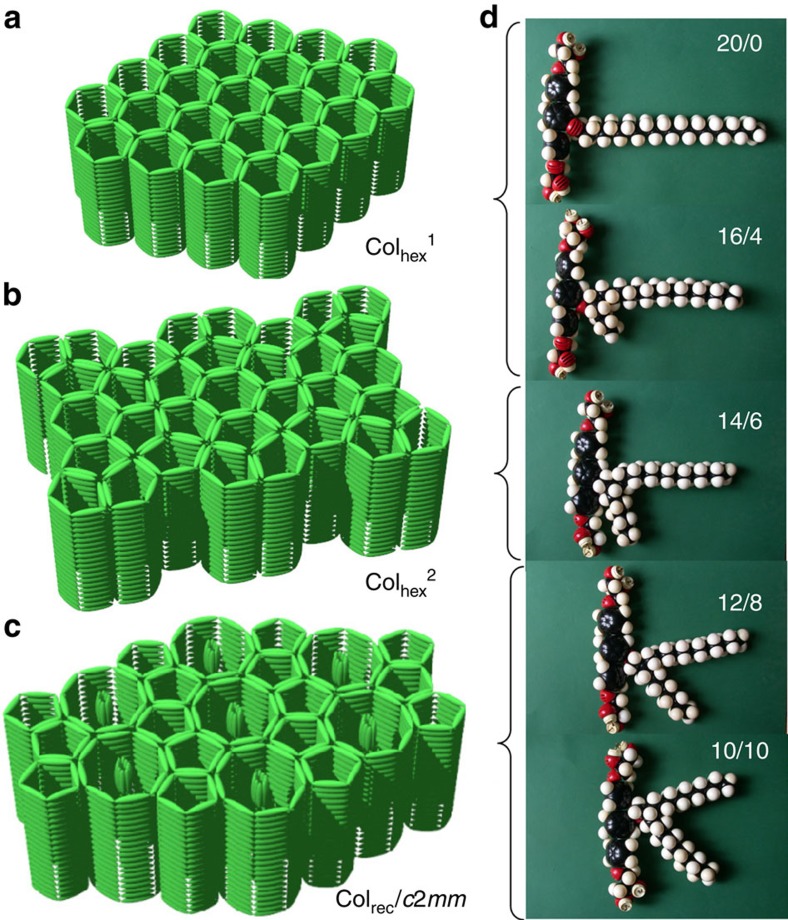
Development of supramolecular organization depending on chain branching. (**a**–**c**) Models showing the organization of the aromatic cores of the molecules (green rods) in the three different LC superstructures (alkyl chains and glycerol groups are omitted); (**d**) molecular models of the corresponding compounds ***m/n***.

Overall, the complex self-assembly achieved in this series of isomeric compounds arises from the decreasing effective chain length at constant chain volume (Fig. 7). The simple hexagonal honeycomb (Fig. 7a), observed for **20/0** and **16/4** is the intrinsically preferred structure based on the ratio of side-chain volume to rod length. Although for all compounds ***m/n*** the length of the side chains is sufficient to reach the centre of the hexagonal channel, increasing chain stretching required for the shorter chains becomes entropically unfavoured. Replacing hexagonal by the smaller pentagonal channels allows the chains to retain high disorder (see Supplementary Note 3), but at the expense of stretching two sides of the pentagons and thus breaking some hydrogen bonds at the apices (Fig. 4d). The remaining hexagons in the complex Col_hex_^2^ structure of **14/6** are believed to serve to partially relieve the frustration of pentagonal packing of a Euclidean plane (Figs 4b and 7b). In the same manner, insertion of hexagons is required in a pentagonally tiled spherical surface as the sphere radius increases (as, for example, in the case of fullerenes). It is worth noting that interesting tiling patterns, including quasiperiodic, have been observed with molecules^13^,^22^ and colloidal particles^14^,^15^,^23^ of a pentagonal symmetry.

Unlike compounds **20/0**, **16/4** and **14/6**, the two compounds **10/10** and **12/8** with shortest chains cannot form the hexagonal channels any more and only layers of fused pentagonal channels remain, thus allowing more chains to be expelled from the tight pentagonal cylinders^24^,^25^. These layers fuse with the formation of octagonal channels. The unreachable void in the centre of the octagonal channel (Fig. 5e) is filled with an additional column consisting of concatenated bundles of rods aligned along the cylinder axis (Fig. 5f,h). The resulting tiling patterns of the LC honeycomb resembles that of inorganic zeolites with the framework-type BIK^26^ in projection viewed along [001] and known, for example, in the alumosilicate *bikitaite*^27^. Furthermore, it represents a unique combination of a honeycomb forming the framework^10^,^11^ and its inverse, the columnar rod-bundle phase^28^, filling the centre of the cells. As the orientations of the molecules in the framework and in the bundles are orthogonal, an unprecedented soft-matter structure is obtained, where for the first time identical π-conjugated rods are aligned parallel as well as perpendicular to the director. This new principle may allow tuning of optical anisotropy and other anisotropic properties^29^,^30^,^31^. It is conceivable, for example, that such structures could be used in sensors or chemical actuators, where a small lattice expansion induced by selected guest species could trigger a transition such as Col_hex_^2^↔Col_rec_/*c*2*mm*, thereby significantly changing properties such as electric permittivity or magnetic permeability.

This work demonstrates that rational design of polyphilic π-conjugated molecules can lead to the emergence of well-ordered nanostructured fluids with reduced symmetry and unparalleled complexity. In comparison, dynamic equilibrium states also determine the type of evolving solid-state structures during solvothermal synthesis of MOFs, COFs and zeolitic materials, often aided by structure-directing agents^32^,^33^. Thus, the knowledge of the basic principles of dynamic self-assembly into complex superstructures gained here should also be beneficial for the rational design of these solid-state materials.

## Methods

### Syntheses

**Figure 8:**
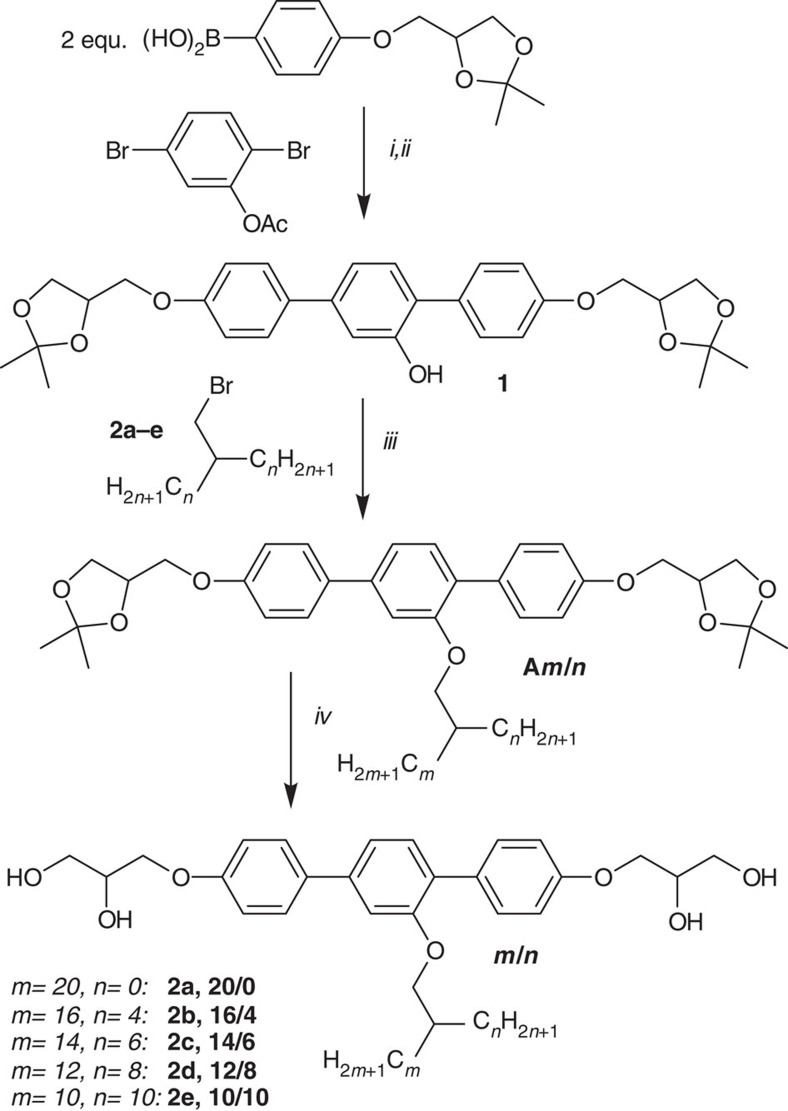
Synthesis of compounds *m/n*. Reagents and conditions: (i) Pd(PPh_3_)_4_, THF, H_2_O, NaHCO_3_, reflux, 12 h; (ii) NaOH, H_2_O, 25 °C, 12 h; (iii) K_2_CO_3_, DMF, 80 °C, 12 h; (iv) CH_3_OH, HCl, reflux 5 h. The synthesis of the starting material *rac*-4-(1,2-isopropylidene-3-glyceryl)benzene boronic acid was performed as reported in ref. 34; **2a** is 1-bromo-*n*-docosane, the syntheses of the branched alkylbromides **2b**–**e** are described in the Supplementary Methods.

All compounds were synthesized in-house according to the synthetic procedure shown in Fig. 8. Details of the experimental procedures and the analytical data of compounds ***m/n*** are given in the accompanying Supplementary Methods^34^.

### Optical and calorimetric investigations

Phase transitions were determined by polarizing microscopy (Leica DMR XP) in conjunction with a heating stage (FP 82 HT, Mettler) and controller (FP 90, Mettler), and by DSC (DSC-7, Perkin Elmer) at heating/cooling rates of 10 K min^−1^ (peak temperatures). Optical investigation was carried out under equilibrium conditions between glass slides, which were used without further treatment; sample thickness was ∼15 μm. A full-wavelength retardation plate was used to determine the sign of birefringence.

### X-ray diffraction

X-ray investigations were carried out using Ni-filtered CuKα radiation (15–30 min exposure time). Aligned samples were obtained on a glass plate. Alignment was achieved on slow cooling (rate: 1–0.01 K min^−1^) of a small droplet of the sample. Alignment is effected at the sample–glass or at the sample–air interface, with domains fibre-like disordered around an axis perpendicular to the interface. The aligned samples were held on a temperature-controlled heating stage and the diffraction patterns were recorded with a 2D detector (Vantec 500, Bruker). High-resolution small-angle powder diffraction experiments were done at beamline I22 at Diamond Light Source. Samples in 1 mm thin-walled borosilicate glass capillaries were held in a modified Linkam hot stage with a cylindrical hole drilled through the silver heating block, the sample area being closed by mica windows at the front and back. A MarCCD 165 detector was used and the sample-to-detector distance was 1.3 m. Samples for GISAX were prepared by melt casting thin films on silicon substrate surface. The films were heated to isotropic liquid in vacuum oven and then slowly cooled. GISAXS measurements were done at Station I16 at Diamond Light Source (U.K.).

### Electron density reconstruction

Fourier reconstruction of the electron density was carried out using the general formula for 2D periodic systems:


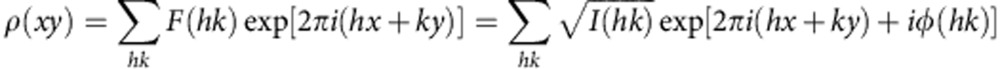


Here, *φ* (*hkl*) is the phase of the (*hkl*) reflection and *I* the corrected intensity. For centrosymmetric structures considered in this work, the phase angle *φ* can only take up the values of 0 or *π*. The choice of phase combination was made on the merit of each reconstructed electron density map obtained using the reflections of strong or medium intensity, as indicated in the Supplementary Tables 3–5. Additional knowledge of the molecules (molecular shape, length, volume of each part and the distribution of electron density among the different moieties) was used in the choice of phase, as explained in the Supplementary Notes 1 and 2, see also Supplementary Figs 8–10.

## Additional information

**How to cite this article:** Poppe, S. *et al*. Zeolite-like liquid crystals. *Nat. Commun.* 6:8637 doi: 10.1038/ncomms9637 (2015).

## Supplementary Material

Supplementary Information Supplementary Figures 1-10, Supplementary Tables 1-6, Supplementary Notes 1-4, Supplementary Methods and Supplementary References

## Figures and Tables

**Figure 1:**
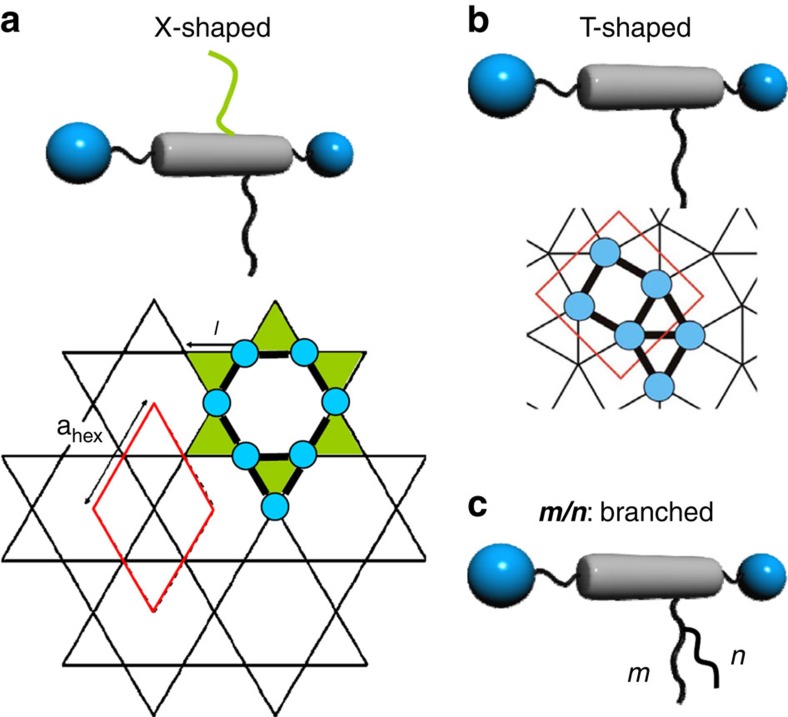
Honeycomb-type LC phases combining channels with different shape. (**a**) Kagome combining honeycomb cells with hexagonal and triangular cross-section as formed by X-shaped molecules having different and incompatible lateral chains at opposite sides of the rod-like core unit^9^,^11^,^16^. (**b**) Archimedean 3^2^.4.3.4 tiling by triangular and square cylinders with ratio 2:1 as found for T-shaped molecules with only one linear chain with certain length^10^. These periodic structures are shown as tiling patterns representing cuts perpendicular to the channels; blue dots represent polar glycerol groups. (**c**) Molecules under consideration herein, having a single branched alkyl chain.

**Table 1:** Transition temperatures (*T*/°C), corresponding enthalpy values (**Δ** *H*/kJ mol^−1^) and lattice parameters (*a,b*/nm) of compounds *m/n* *.

**Compouds**	***m***	***n***	**Phase transitions**	***a,b***	**Channel cross-sections**
**20/0**	20	0	Cr 35 [35.1] Col_hex_^1^/*p*6*mm* 185 [6.7] Iso	*a*_hex_=4.2 nm	Hexagons
**16/4**	16	4	Cr 43 [6.7] Col_hex_^1^/*p*6*mm* 144 [4.5] Iso	*a*_hex_=4.2 nm	Hexagons
**14/6**	14	6	Cr 53 [7.3] M_1_ ∼130 [<0.01] Col_hex_^2^/*p*6*mm* 145 [4.0] Iso	*a*_hex_=9.7 nm	Pentagons+hexagons
**12/8**	12	8	Cr_1_ 72 [7.1] Cr_2_ 94 [6.9] Col_rec_/*c*2*mm* 146 [4.2] Iso	*a*=6.2 nm *b*=15.5 nm	Pentagons+octagons
**10/10**	10	10	Cr 75 [7.1] Col_rec_/*c*2*mm* 147 [4.5] Iso	*a*=6.4 nm *b*=16.1 nm	Pentagons+octagons

Cr, crystalline solid; Iso, isotropic liquid; M_1_, unknown liquid crystal; Col_hex_^1^, Col_hex_^2^, Col_rec_, columnar liquid crystal with hexagonal and rectangular two-dimensional lattice, respectively.

For differential scanning calorimetry traces, see Supplementary Fig. 1; for tables with X-ray diffraction data, see Supplementary Tables 1–5.

^*^First heating at 10 K min^−1^, peak temperatures were used; transition enthalpies in square brackets.
