# Supplementary material for: Zeolite-like liquid crystals
Source: Nat Commun. 2015 Oct 21;6:8637. doi: 10.1038/ncomms9637 (PMC4639914; doi:10.1038/ncomms9637)
Supplement: Supplementary Information — Supplementary Figures 1-10, Supplementary Tables 1-6, Supplementary Notes 1-4, Supplementary Methods and Supplementary References [file ncomms9637-s1.pdf]

Supplementary Figures

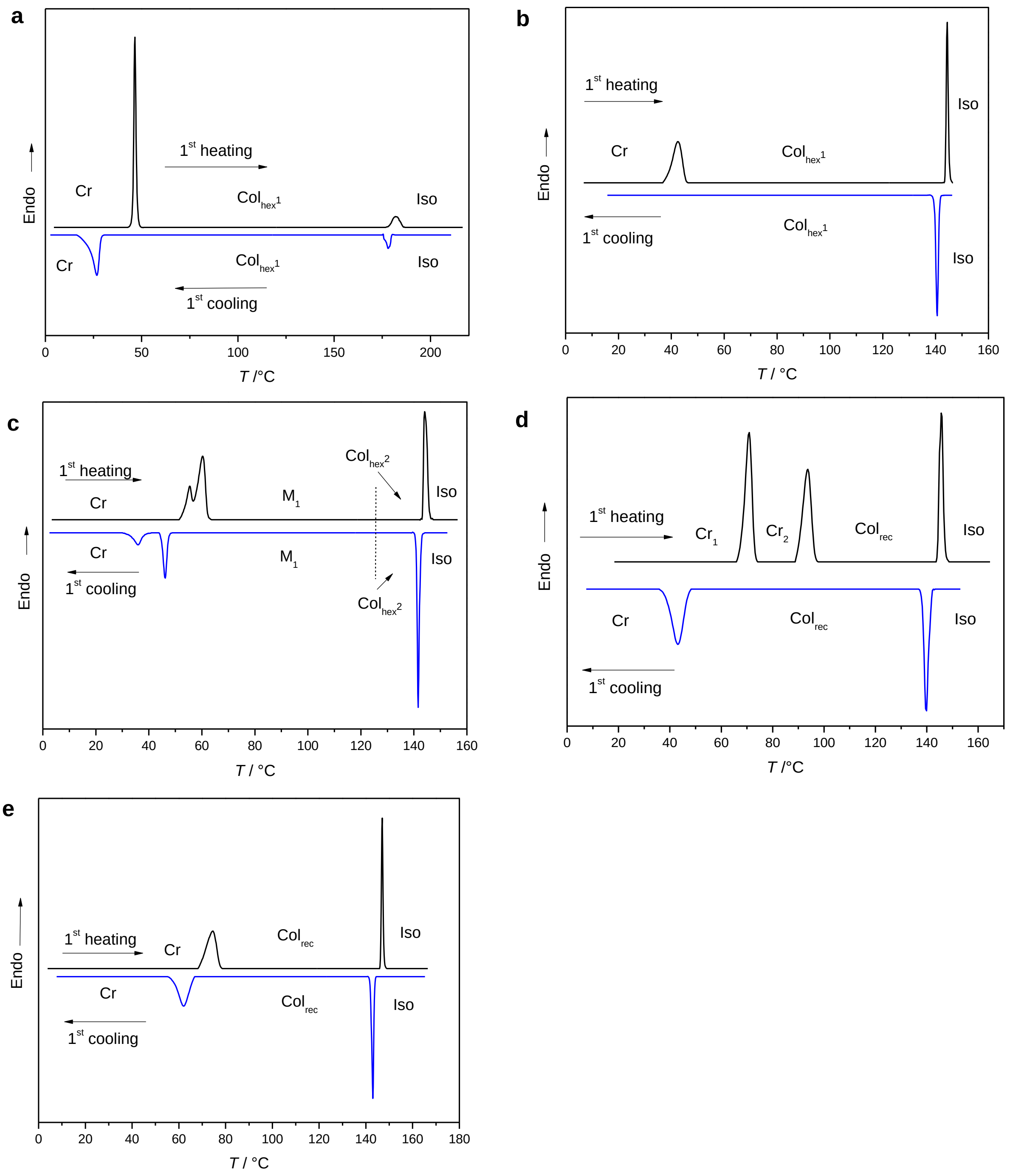

Supplementary Figure 1 │Differential scanning calorimetry. DSC traces of compounds (**a**) **20/0**, (**b**) **16/4**, (**c**) **14/6**, (**d**) **12/8**, and (**e**) **10/10**.

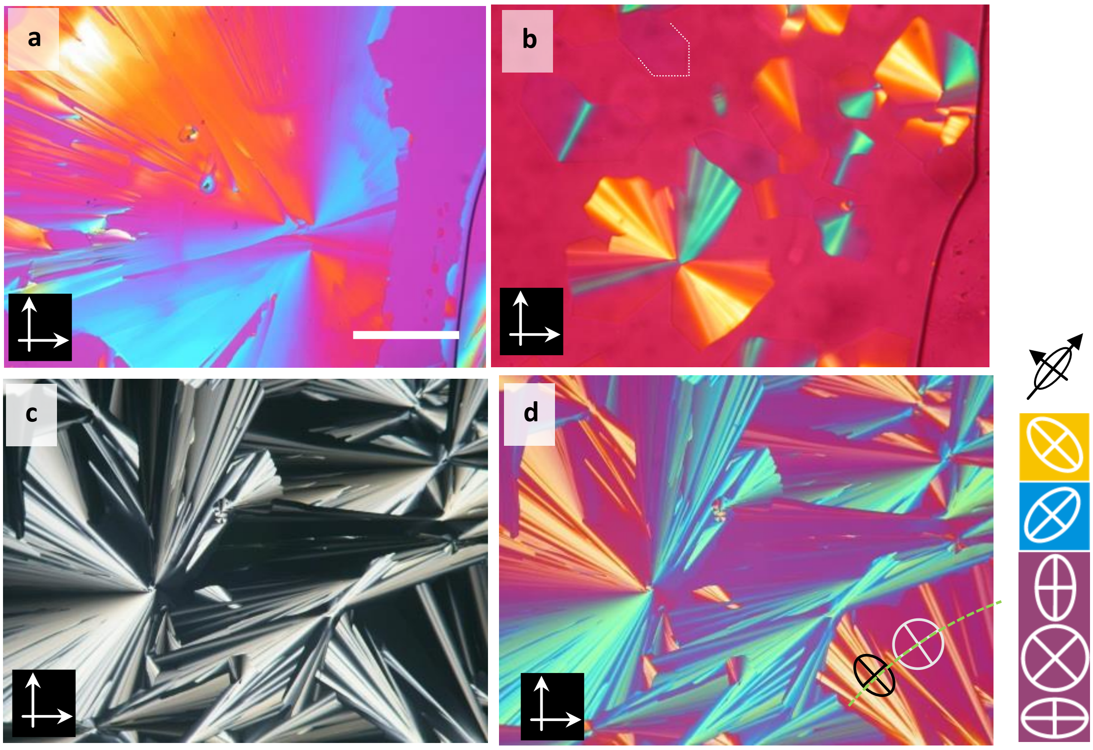

Supplementary Figure 2 │Textures observed between crossed polarizers. (**a**) Col_{hex}^{1}/*p*6*mm* phase of **16/4** *T* = at 120 °C; (**b**) Col_{hex}^{2}/*p*6*mm* phase of **14/6** at *T* = 145 °C during growth from Iso (see also the hexagonal shape of the growing nearly homeotropic domains); (**c,d**) Col_{rec}/*c*2*mm* phase of **12/8** at *T* = 140 °C; textures were observed between non-treated glass plates; the textures in (**a,b**) and (**d**) were observed with additional λ-retarder plate. The orientation of the indicatrix of the λ-plate (top) and the distinct orientations of the optical axes in the differently colored areas of the textures are shown at the right. In the textures in (**c,d**) we see two colours in each sector of the fans: yellow and magenta in southeast-northwest fans and blue and magenta in SW-NE fans. Scale bar in a) is 100 μm and refers to all figures. Considering the two orientations, (10) and (11), revealed by the GISAXS experiments (see Fig. 6), we associate the two colours with those two orientations of the unit cell, in both cases the columns lying in the film plane. The dashed curve in (**d**) indicates the trajectory of the columns, with the projections of the indicatrix sketched on it in the yellow and magenta regions. For a substantial portion of the fan, even where the column direction is around 45° to the polarizers, the birefringence is virtually zero (magenta colour, circular cut through indicatrix). That means that the indicatrix of the *c*2*mm* phase, that should in general be biaxial, is in this particular case uniaxial or close to it, but with the optic axis being along either the [100] or the [110] direction, i.e. perpendicular rather than parallel to the column axis. This unusual situation appears to be another consequence of the hybrid transverse/axial orientation of the mesogens in the zeolite-like *c*2*mm* phase. Without the axial molecules this would be impossible in case of planar orientation of the columns. If all molecules were transverse, birefringence would have always been negative, no matter on which plane the columns were lying.

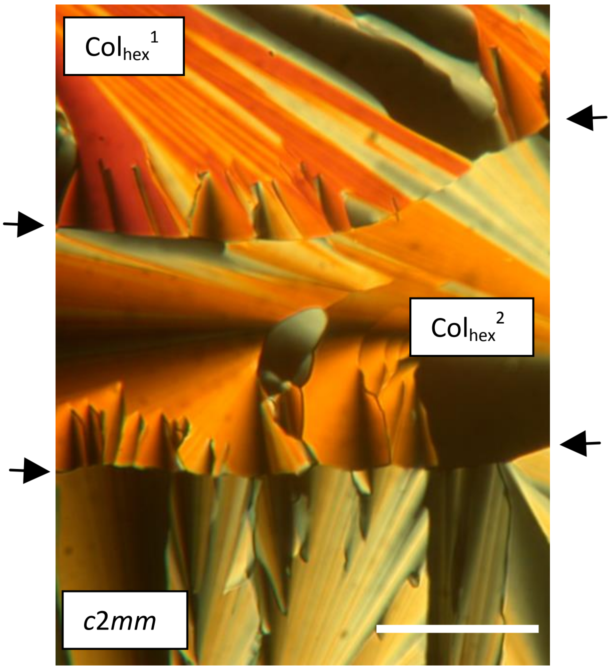

Supplementary Figure 3 │ Induced Col$_{hex}$^{2} phase. Contact region between the low birefringent *c*2*mm* phase of **12/8** (bottom) and the high birefringent Col$_{hex}$^{1} phase of compound **16/4** (top) with induced Col$_{hex}$^{2} phase between them as observed between crossed polarizers at T = 136 °C; arrows indicate the positions of the phase boundaries; scale bar is 100 µm.

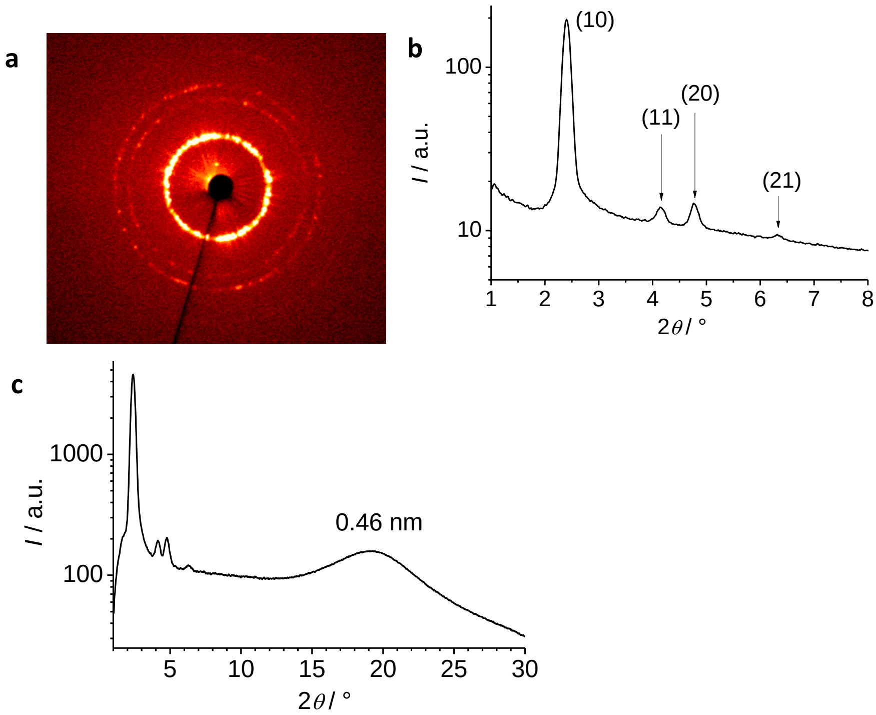

Supplementary Figure 4 │ XRD data for compound 20/0. (**a**) X-ray diffraction pattern at T = 160 °C; (**b**) θ-scan of the small angle region of the diffraction pattern of the Col$_{hex}$^{1}/*p*6*mm* phase; (**c**) θ-scan of the wide angle region of the diffraction pattern with the *d* value for the maximum of the diffuse outer scattering.

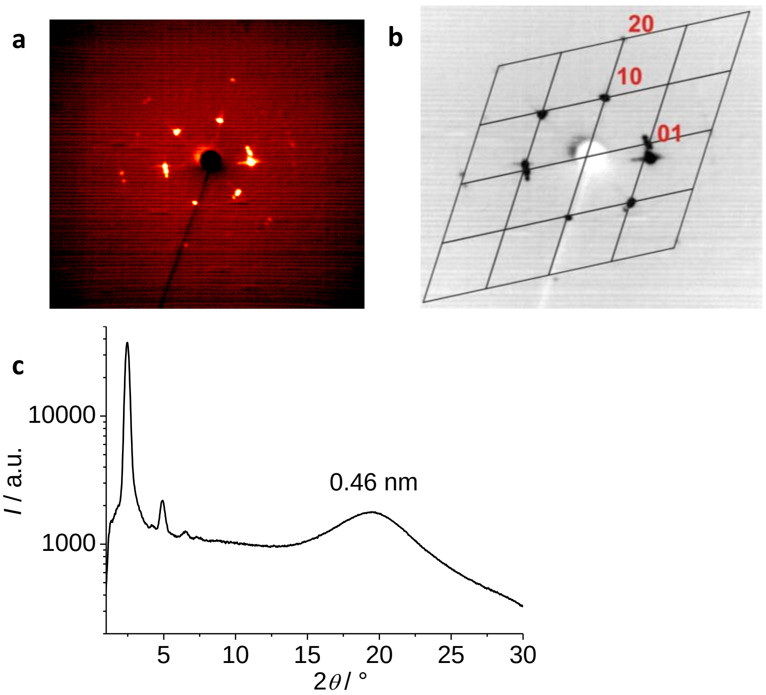

Supplementary Figure 5 │ XRD data for compound 16/4. (**a**) X-ray diffraction pattern at 135 °C; (**b**) indexing of the small angle reflexes; (**c**) θ-scan of the wide angle region of the diffraction pattern with the d value for the maximum of the diffuse outer scattering.

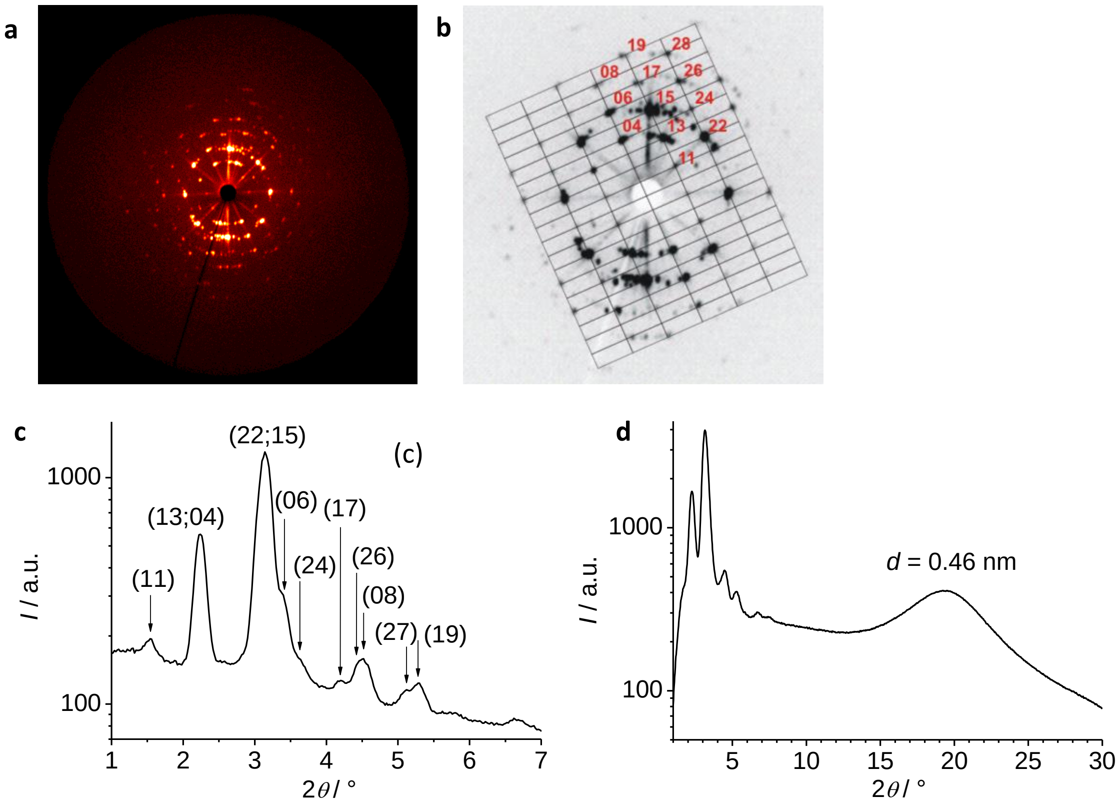

Supplementary Figure 6 │ XRD data for compound 12/8. (**a**) X-ray diffraction pattern of the Col_{rec}/$c2mm$ phase at 135 °C; (**b**) indexing of the small angle reflexes; (**c**) θ-scan of the small angle region of the diffraction pattern of the Col_{rec} / $c2mm$ phase; (**d**) θ-scan of the wide angle region of the diffraction pattern with the d value for the maximum of the diffuse outer scattering.

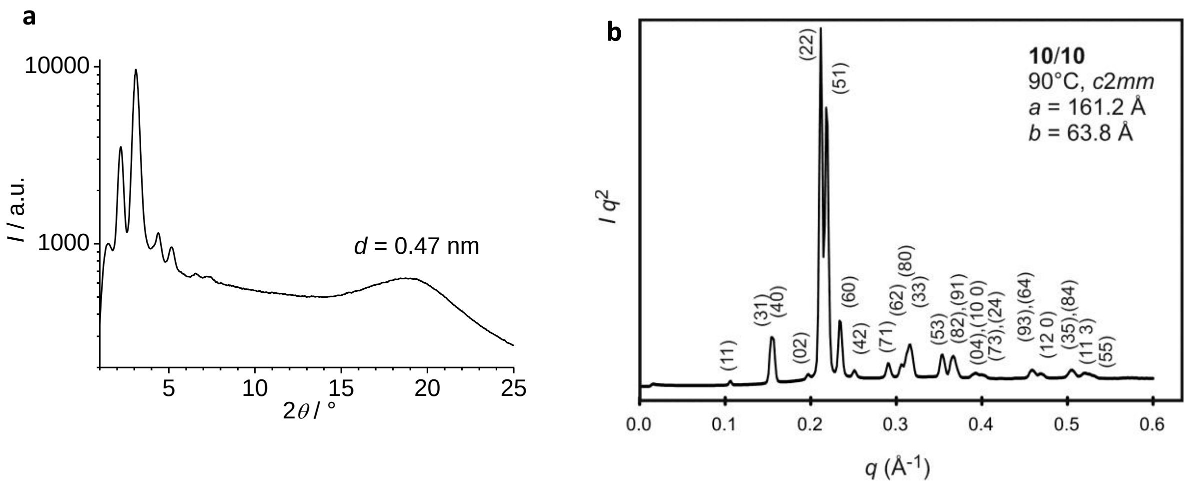

Supplementary Figure 7 │XRD patterns of the Col$_{rec}$/*c*2*mm* phase of compound 10/10. (**a**) θ-scan of the wide angle region of the diffraction pattern with the *d* value for the maximum of the diffuse outer scattering at 140 °C; (**b**) diffraction pattern of the Col$_{rec}$/*c*2*mm* phase at T = 90 °C as obtained with a synchrotron source.

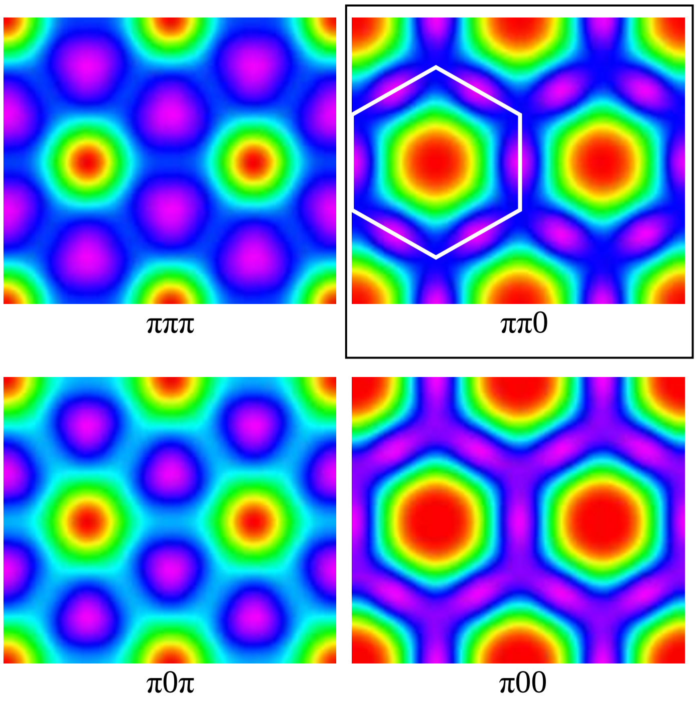

Supplementary Figure 8 │ Reconstructed electron density maps of the Col$_{hex}{}^{1}$/*p*6*mm* phase of compound 16/4. Structure factor combinations based on the three reflections (10), (11) and (20) (see Supplementary Table 3); electron density color code: purple/blue = high, red/yellow = low, green = medium. Based on the electron density maps, the negative birefringence of this phase and the molecular dimensions, only the hexagonal honeycomb structures (white line) is possible for the Col$_{hex}{}^{1}$ phase.

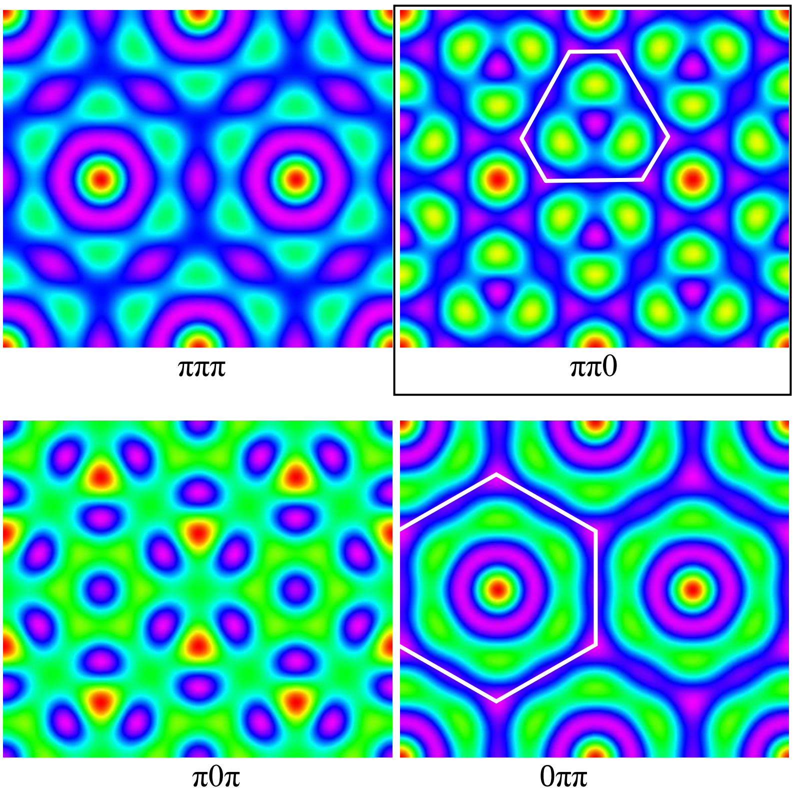

Supplementary Figure 9 │ Reconstructed electron density maps of the Col$_{hex}^{2}$/*p*6*mm* phase of compound 14/6. Different structure factor combinations based on the three strongest reflections (11), (21) and (30) were used (see Supplementary Table 4); electron density color code: purple/blue = high, red/yellow = low, green = medium; the selected phase combination is framed; see Supplementary Note 1 for selection of phase combination.

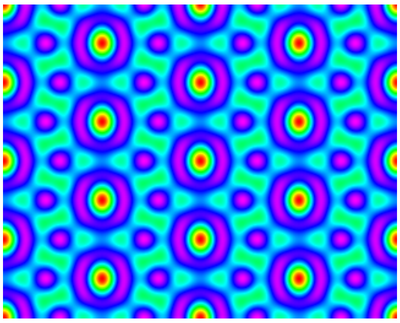
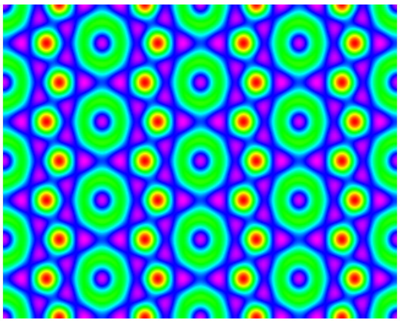
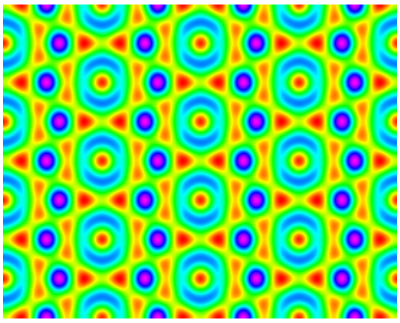

(**a**) πππππππ (**b**) ππππ000 (**c**) ππ0ππ0π

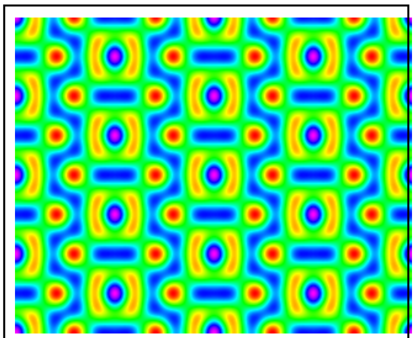
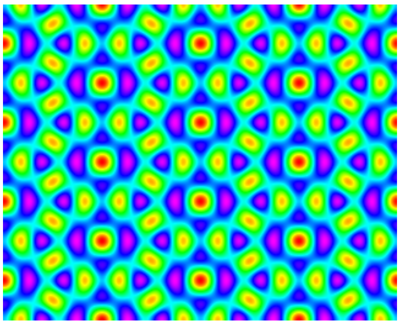
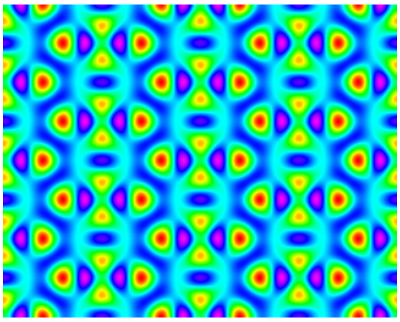

(**d**) ππ00000 (**e**) π0π0π00 (**f**) π0π00ππ

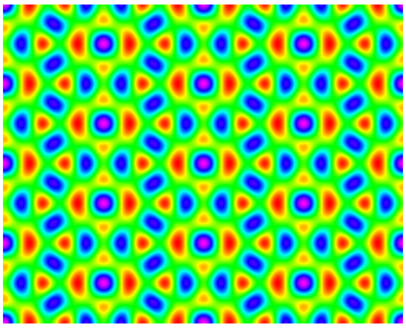
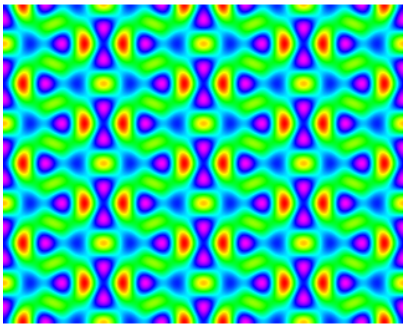

(**g**) π00π00π (**h**) π000π00

Supplementary Figure 10 │ Eight essentially different electron density maps for the *c*2*mm* phase of 10/10. Reconstructed from the small-angle diffraction intensities measured from the diffractogram in Supplementary Fig. 7**b** and listed in Supplementary Table 5. The chosen map is (**d**); see Supplementary Note 2 for selection of phase combination.

Supplementary Tables

Supplementary Table 1 │ Experimental and calculated *d*-spacings of the observed SAXS reflections of the hexagonal columnar phase $Col_{hex}^{1}/p6mm$ of compound 20/0 at 160 °C.

2θ	θ	*d*-value	*hk*	d_{calc}	d_{obs}-d_{calc}
2.403	1.202	3.676	10	3.672	0.00
4.149	2.075	2.130	11	2.120	0.01
4.775	2.388	1.851	20	1.836	0.02
6.312	3.156	1.400	21	1.388	0.01
19.210	9.605	0.462	diff		
Col_{hex}: a = 4.24 nm					

Supplementary Table 2 │ Experimental and calculated *d*-spacings of the observed SAXS reflections of the rectangular columnar phase $Col_{rec}/c2mm$ of compound 12/8 at 135 °C.

2θ	θ	*d*-value	*hk*	d_{calc}	d_{obs}-d_{calc}
1.530	0.765	5.774	11	5.773	0.00
2.222	1.111	3.976	13	3.976	0.00
2.262	1.131	3.906	04	3.877	0.03
3.092	1.546	2.857	22	2.887	-0.03
3.165	1.583	2.791	15	2.776	0.02
3.391	1.696	2.605	06	2.585	0.02
3.637	1.819	2.429	24	2.426	0.00
4.202	2.101	2.103	17	2.087	0.02
4.420	2.210	1.999	26	1.988	0.01
4.518	2.259	1.956	08	1.939	0.02
5.277	2.639	1.675	19	1.661	0.01
5.331	2.666	1.658	28	1.645	0.01
19.43	9.715	0.457	diff		
Col_{rec} : a = 6.22 nm; b = 15.51 nm					

Supplementary Table 3 │ Indices, measured and calculated *d*-spacings, intensities, multiplicities and phase angles of diffraction peaks used for reconstruction of electron density map of the $Col_{hex}^{1}/p6mm$ phase of compound 16/4 at 135 °C.[a]

(*hk*)	θ/°	$d_{obs.}$/nm	$d_{cal.}$/nm	intensity	multiplicity	ϕ
(10)	1.230	3.593	3.594	100	6	π
(11)	2.137	2.068	2.075	0.4	6	π
(20)	2.450	1.803	1.797	5.4	6	0
(21)	3.241	1.364	1.358	0.9	12	-
(30)	3.675	1.203	1.198	0.2	6	-
diff	9.760	0.455	-	-	-	-
Col_{hex}: a = 4.15 nm						

[a] The intensities are normalized to that of the (10) diffraction peak.

Supplementary Table 4 │ Indices, measured and calculated *d*-spacings, intensities, multiplicities and phase angles of diffraction peaks used for reconstruction of electron density map of the Col_{hex}^{2}/*p*6*mm* phase of compound 14/6 at 135 °C. [a]

(*hk*)	*θ*/°	$d_{obs.}$/nm	$d_{cal.}$/nm	intensity	multiplicity	ϕ
(11)	0.909	4.862	4.862	13.2	6	π
(21)	1.381	3.200	3.183	100	12	π
(30)	1.554	2.843	2.807	14.3	6	0
(22)	1.827	2.418	2.431	1.7	6	-
(31)	1.887	2.341	2.336	0.6	12	-
(32)	2.269	1.947	1.932	1.5	12	-
(50)	2.613	1.691	1.684	0.8	6	-
(33)	2.701	1.636	1.621	1.1	6	-
(51)	2.896	1.526	1.512	0.6	12	-
diff	9.700	0.458	-	-	-	-
Col_{hex}: *a* = 9.72 nm						

[a] The intensities are normalized to that of the (21) diffraction peak.

Supplementary Table 5 │Diffraction peaks of compound 10/10 at 90°C: indices, measured and calculated *d*-spacings, intensities, multiplicities and phase angles used for the reconstruction of the electron density map.[a]

(*hk*)	d_{obs}	d_{calc}	Intensity	multiplicity	ϕ
(11)	59.23	59.32	4.0	4	π
(31)	41.07	41.09	15.0	4	π
(40)	40.28	40.29	22.7	2	0
(02)	31.87	31.90	0.8	2	0
(22)	29.70	29.66	90.7	4	0
(51)	28.76	28.77	100	4	0
(60)	26.79	26.86	16.2	2	0
(42)	25.06	25.01	1.0	4	-
(71)	21.61	21.66	3.1	4	-
(62)	20.52	20.55	1.4	4	-
(80)	20.07	20.15	5.6	2	-
(33)	19.80	19.77	3.8	4	-
(53)	17.76	17.75	3.6	4	-
(82)<br>(91)	17.14	17.03<br>17.24	3.9	4<br>4	-
(04)<br>(10 0)	16.00	15.95<br>16.12	1.4	2<br>2	-
(73)<br>(24)	15.60	15.62<br>15.65	0.3	4<br>4	-
(93)<br>(64)	13.69	13.70<br>13.71	0.9	4<br>4	-
(12 0)	13.39	13.43	0.5	2	-
(35)<br>(84)	12.44	12.41<br>12.50	0.9	4<br>4	-
(11 3)	12.10	12.06	0.4	4	-
(55)	11.92	11.86	0.4	4	-

[a] The best-fit unit cell parameters are *a* = 161.16 Å and *b* = 63.80 Å, with plane group *c*2*mm*.

Supplementary Table 6 │ Calculation of the molecular volume (V_{mol}), volume of the hypothetical unit cells (V_{cell}) the number of molecules in these unit cells (n_{cell}) and other structural parameters.[a]

Comp.	*a*, *b*/nm	V_{cell}/nm^3	V_{mol}/nm^3	n_{cryst}	n_{liq}	n_{cell}	n_{wall}	n_{bundle}
**20/0**	4.24	7.16	1.07	6.69	5.26	5.97	2.0	
**16/4**	4.15	6.71	1.07	6.27	4.93	5.60	1.9	
**14/6**	9.72	37.64	1.07	35.18	27.64	31.41	1.7	
**12/8**	6.22;15.51	44.37	1.07	41.47	32.58	37.02	1.9	7.0
**10/10**	6.38;16.12	46.28	1.07	43.25	33.98	38.61	1.9	11.0

[a] V_{cell} = volume of the unit cell defined by a x b x 0.46 nm for rectangular columnar phases and a^2 x sin(60°) x 0.45 nm for hexagonal phase of **16/4** and a^2 x sin(60°) x 0.46 for the hexagonal phases of **14/6** and **20/0** (value chosen for h corresponds to the position of the diffuse wide angle scattering maximum in the XRD patterns, see insets in Figs. 3**a** and 4**a**, and Supplementary Figs. 4-7); V_{mol} = molecular volume as calculated using crystal volume increments *(14)*, the volume of the chains is 0.56 nm^3 and that of the aromatic cores including the glycerols and the ether oxygens is 0.51 nm^3; n_{cryst} = number of molecules in the unit cell, calculated according to $n_{cell} = V_{cell}/V_{mol}$ (average packing coefficient in the crystal is k = 0.7); n_{liqu} = number of molecules in the unit cell of an isotropic liquid with an average packing coefficient k = 0.55, calculated according to n_{liqu} = 0.55/0.7 x n_{cryst}; n_{cell} = number of molecules in the unit cell in the LC phase estimated as the average of that in the n_{cryst} and n_{liqu}; n_{wall} = number of molecules in the cross section of the cylinder walls as calculated from n_{cell}/n_{sides}; n_{bundle} = number of molecules in the cross section of the coaxial bundles in the octagonal cells of the *c*2*mm* phase, calculated as described in the Supplementary Note 4.

Supplementary Notes

Supplementary Note 1 │ Selection of the phase combination for the Col_{hex}^2 phase of compound 14/6. Based on the electron density maps (Supplementary Fig. 9) in principle two different structures are possible for the Col_{hex}^2 phase with negative birefringence (having the aromatic cores organize in the plane of the 2D lattice), one with giant cylinders formed by two molecules along each of the walls of the hexagons (12-hexagons, see 0ππ); the other one is the hexagon/pentagon tiling as shown in Fig. 4**b-d** in the main text. The former is discarded as the side length of the 12-hexagons would be 5.7 nm, which is significantly more than twice the molecular length (2 x 2.45 = 4.9 nm). In comparison, the length of one of the longer walls of the 9-hexagons in the pentagon/hexagon structure is 4.85 nm, which is rather close to the required 2 x 2.45 = 4.9 nm. Also the honeycomb walls of the small 6-hexagons of the pentagon/hexagon tiling have a length of 2.4 nm, in very good agreement with L_{mol}. If the giant hexagonal structure was assumed, then the electron density map 0ππ indicates the presence of an additional high electron density cylinder (purple) in the centre of each 12-hexagon. In principle these cylinders could be formed by coaxial rod-bundles, as it is the case of the Col_{rec}/*c*2*mm* phase (the rod-bundle ribbons would in this case form a hollow cylinder, filled with the alkyl chains of the inner molecules). However, this would lead to a significant reduction of the birefringence or could even lead to positive birefringence, which is in conflict with the negative birefringence of the Col_{hex}^2 phase, being very similar to the birefringence of the Col_{hex}^1 phase (see Fig. 5**c** and Supplementary Figs. 2b and 3). Therefore, the (ππ0) phase combination, best reflecting the molecular dimensions and being in agreement with the distribution of electron rich (aromatics, glycerols; blue/purple) and electron poor (alkyl chains; red/yellow/green) building blocks, was selected.

Supplementary Note 2 │ Selection of the phase combination for the Col_{rec}/*c*2*mm* phase of compound 10/10. Even though 7 diffraction peaks have been used for the reconstruction of the *c*2*mm* electron density maps, two of them, (11) and (02), are very weak and do not make much qualitative difference to the maps reconstructed (Supplementary Fig. 7b and Supplementary Table 5). Certain phase combinations show essentially the same map, with

only the origin of the unit cell shifted to four different positions due to the *c*2*mm* symmetry. The number of essentially different maps obtained is in fact eight, and they are shown in the Supplementary Fig. 10. To determine the best phase combination, the first criterion used is that the map should have distinctive high density and low density regions, which can be attributed to the rigid aromatic backbone and flexible side chains respectively. On this basis maps (**a**), (**b**) and (**h**) can be eliminated, as there are large medium density (green, light-blue) regions which cannot be explained by a packing of **10**/**10** molecules. Next, map (**f**) can also be dismissed as the relative volume of the high density regions (blue to purple) is too high to be attributed to aromatic backbones, although if it was selected it would have given the same molecular arrangement as the chosen map (**d**). For the remaining four maps, maps (**c**) and (**g**) are all full of isolated high-density dots and it is difficult to see any honeycomb network. In addition, in (**c**) the aromatic backbone form a nearly circular cell which is rather unreasonable. Both (**c**) and (**e**) suggest models where the side length of the honeycombs is much longer than that of the molecular backbone. Between maps (**d**) and (**e**), (**d**) is better in that the high and low density regions are more uniform. Also the structure based on map (**e**) would have to have structural elements significantly longer than the length of the molecular core. While such electron density maps have their limitations due to the finite number of Fourier terms, map (**d**) is chosen in the end as on its basis an almost continuous honeycomb framework can be constructed, with wall lengths matching the molecular lengths, and because of its general agreement with other physical data, such as the molecular core length, the two orientations of the 2D lattice observed by GISAXS (see Fig. 6), and the reduced absolute value of birefringence, as discussed in the main text.

Supplementary Note 3 │ Molecular organization in the Col$_{hex}^{2}$ phase. As shown in Supplementary Fig. 11 there is a overcrowding of the pentagonal channels whereas sufficient space is available for the alkyl chains in the hexagonal channels. Supplementary Fig. 12 shows the calculation of the side-length ratio of the pentagons in the Col$_{hex}^{2}$ phase.

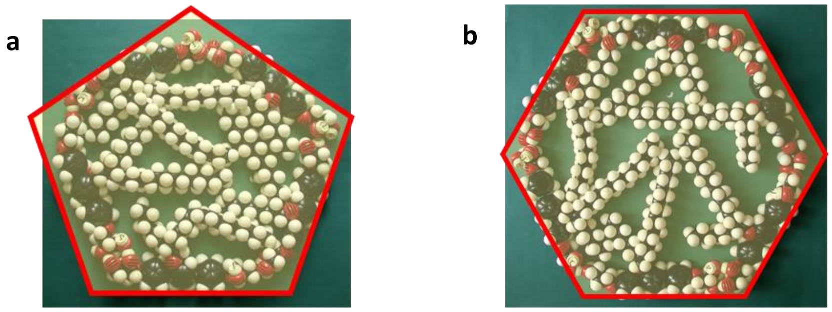

Supplementary Figure 11 │ Polygons in the Col$_{hex}^{2}$/*p*6*mm* phase of 14/6. (**a**) the pentagons are sterically overcrowded; (**b**) the space in the hexagons is filled, but the chains have to be stretched to reach the center.

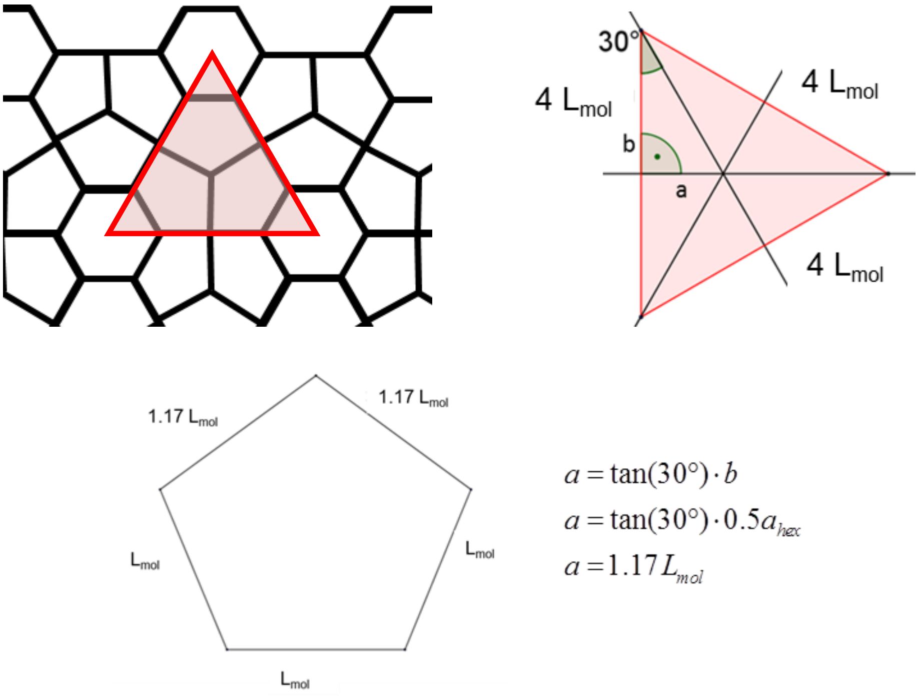

Supplementary Figure 12 │ Calculation of the side length ratio of the pentagon in the Col_{hex}^{2}/*p*6*mm* phase.

Supplementary Note 4 │ Estimation of the number of molecules in the honeycomb walls and in the coaxial bundles of the Col_{rec}/*c*2*mm* phase. For development of structural models the number of molecules in a unit cell defined by the lattice parameters and a height (h) of h = 0.45 or 0.46 nm (maximum of the diffuse wide angle scattering) was calculated (V_{cell}) and divided by the molecular volume (V_{mol}), thus giving the number of molecules per unit cell (n_{cell}), see Supplementary Table 6. Assuming the proposed tiling pattern the number of cylinder side walls (n_{sides}) per unit cell is calculated, leading to n_{sides} = 3 for the Col_{hex}^{1} phase, n_{sides} = 18 for the Col_{hex}^{2} phase and n_{sides} = 18 for the Col_{rec}/*c*2*mm* phase, respectively. The number of molecules arranged back-to-back across the cylinder walls (n_{wall}) is calculated according to n_{cell}/n_{sides}, leading to n_{wall} = 1.9…2.0 for the Col_{hex}^{1} phases and n_{wall} = 1.7 for the Col_{hex}^{2} phase. n_{wall} is not necessarily an integer number, as the considered systems are fluid and there are no fixed positions of the molecules. Hence, it is an average number considering the dynamics of the system and involving some defects and staggering of the molecules. The slightly reduced number of n_{wall} = 1.7 for the Col_{hex}^{2} phase might be due to the three less defined walls in each 9-hexagon.

For the Col_{rec}/*c*2*mm* phase the presence of the axial rod-bundles must be considered. The number of molecules in the cross-section of these additional bundles can be estimated by assuming that n_{wall} of the honeycomb framework is the same as in Col_{hex}^{1} (n_{wall} = 1.9). Thus the 18 cylinder walls per unit cell require 18 x 1.9 = 34.2 molecules, leaving ~2.8 and ~4.4 molecules per unit cell for these coaxial bundles (n_{cell} – 34.2) in the *c*2*mm* phases of **12/8** and **10/10**, respectively. As the coaxial molecules contribute to more than just one unit cell, their contribution to each unit cell can be calculated by dividing the height of the unit cell (h) by the molecular length (L_{mol} = 2.45 nm), leading to about 0.2. This means that these coaxial molecules contribute with 20% to each of 5 adjacent unit cells. Thus, there are 14 and 22 molecules contributing to the rod-bundles in each unit cell of **12/8** and **10/10**, respectively. Because there are two coaxial bundles per unit cell the number of molecules arranged side by side in the bundles (n_{bundle}) is half this number, i.e. n_{bundle} = 7 and 11, respectively (see Supplementary Table 6). This number is very similar to that found in the cross section of the

axial bundles (~ 10 molecules) in the hexagonal axial bundle phases without additional honeycomb framework. *(7)*

Supplementary Methods

Synthesis of the compounds was performed according to Fig. 8 using the following procedures. Unless otherwise noted, all starting materials were purchased from commercial sources and were used without further purification. Column chromatography was performed with silica gel 60 (63-200 µm, Fluka). Determination of structures and purity of intermediates and products was obtained by NMR spectroscopy (VARIAN Gemini 2000 and Unity Inova 500, all spectra were recorded at 27 °C). Microanalyses were performed using a CARLO Erba-CHNO 1102 elemental analyzer and a micrOTOF HR-ESI mass spectrometer (Bruker). The purity of all products was checked with thin layer chromatography (silicagel 60 F_{254}, Merck). $CHCl_3$/EtOAc mixtures and $CHCl_3$/MeOH mixtures were used as eluents and the spots were detected by UV radiation. All branched compounds with unequal chain length represent racemic mixtures; C_mH_{2m+1} and C_nH_{2n+1} represent linear chains.

4,4''-Bis(1,2-isopropylidene-3-glyceryl)-*p*-terphenyl-2'-ol (1). Compound **1** was synthesized using the Suzuki cross coupling method *(1)*. A mixture of 1,4-dibromophenylacetate (*2,3*) (2.64 g, 10.0 mmol), 4-(1,2-isopropylidene-3-glyceryl)benzene boronic acid (*4,5*) (6.8 g, 22.0 mmol), [Pd(PPh_3)$_4$] (0.7 g, 0.5 mmol), THF (150 mL) and sat. aq. $NaHCO_3$ (100 mL) was stirred under an argon atmosphere at reflux for 12 h. After cooling to room temperature NaOH (3.7 g, 90.0 mmol) was added and the reaction was allowed to cool overnight. The mixture was extracted with $CHCl_3$ (3 x 50 mL). The combined organic layers were washed with water and brine. After drying over anhydrous Na_2SO_4, the solvent was removed under reduced pressure. The residue was purified by column chromatography ($CHCl_3$/EtOAc, 4/1 v/v) and crystallization from MeOH. Colorless solid; Yield: 2.0 g (40 %); mp. 139 °C ; TLC ($CHCl_3$:EtOAc, 4:1 v/v): R_f = 0.50; ^{1}H-NMR ($CDCl_3$, 500 MHz): δ / ppm = 7.53 (d, $^3J_{H,H}$ = 8.7 Hz, 2 H, Aryl-*H*), 7.40 (d, $^3J_{H,H}$ = 8.9 Hz, 2 H, Aryl-*H*), 7.23 (d, $^3J_{H,H}$ = 8.3 Hz, 1 H, Aryl-*H*), 7.15 (m, 2 H, Aryl-*H*), 7.03 (d, $^3J_{H,H}$ = 8.6 Hz, 2 H, Aryl-*H*), 6.97 (d, $^3J_{H,H}$ = 9.1 Hz, 2 H, Aryl-*H*), 5.16 (s, 1 H, O*H*), 4.48 (m, 2 H, OC*H*), 4.18 (m, 2 H, OCH_2), 4.09 (m, 2 H, OCH_2), 3.98 (m, 2 H, OCH_2), 3.90 (m, 2 H, OCH_2), 1.46 (s, 6 H, CH_3), 1.39 (s, 6 H, CH_3).

Bromoalkanes (2a-e). 1-Bromo-*n*-docosane (**2a**) was obtained from commercial sources and was used as received. The branched alkyl bromides **2b-e** were synthesized as described in more detail below by alkylation of dialkyl malonates (methyl, ethyl) **3b-e**, followed by dealkoxycarbonylation, yielding methyl or ethyl 2-alkylalkanoates **4b-e**; reduction of **4b-e** to the 2-alkylalkanols **5b-e** with $LiAlH_4$ and transformation into the 2-alkyl-1-bromo-alkanes **2b-e** with aqu. HBr/conc. H_2SO_4.

Dialkylated malonates 3b-e *(6,7)*. Diethyl 2-butyl malonate was obtained from Sigma Aldrich and was used as obtained. The others were synthesized according to the following procedures.

Procedure A: The reaction was carried out under an argon atmosphere. Sodium hydride (1.5 eq / 60% in mineral oil) was slowly suspended in DMF (abs., 100 mL) and the mixture was cooled to 0 °C. Dialkyl malonate (1 eq.) and the appropriate 1-bromoalkane (0.8eq for monoalkylation) in DMF (50 mL) was added one after another and the mixture was stirred at room temperature for 3 h. After reaction water (250 mL) was added and the mixture was extracted with diethyl ether (3 x 100 mL). The combined organic layers were washed with sat.

aqu. LiCl, water and brine. After drying over anhydrous Na_2SO_4 the solvent was removed under reduced pressure. The residue was purified by column chromatography (1. *n*-hexane, 2. $CHCl_3$).

Procedure B: The reaction was carried out under an argon atmosphere. Sodium (3 eq) was dissolved slowly in MeOH (abs., 50 mL / g sodium); dialkyl malonate (1eq) and 1-bromoalkane (1.1 eq for monoalkylation, 2.2 eq for dialkylation) were added one after another. The reaction was stirred at room temperature for 8 h. The mixture was concentrated under reduced pressure and poured into ice water to dissolve the precipitate. After extraction with diethyl ether (3 x 100 mL) the combined organic layers were washed with water and brine. After drying over anhydrous Na_2SO_4 the solvent was removed under reduced pressure. The residue was purified by column chromatography (1. *n*-hexane, 2. $CHCl_3$).

Dimethyl 2-butyl-2-hexadecylmalonate 3b. Synthesized from diethyl 2-butylmalonate (20.00 g, 0.09 mol), 1-bromohexadecane (31.03 g, 0.10 mol) and sodium (10.60 g, 0.46 mol) in methanol (abs., 150 mL) *(7)*. Colorless liquid; Yield: 25.24 g (66%); TLC (*n*-hexane): R_f = 0.10; ^{1}H-NMR ($CDCl_3$, 400 MHz): δ / ppm = 3.68 (s, 6H, OCH_3), 1.83 (m, 4H, CH(CH_2)$_2$), 1.23 (m, 32H, CH_2), 0.85 (m, 6H, CH_3).

Diethyl 2-hexyl-2-tetradecylmalonate 3c. Diethyl 2-hexylmalonate *(8)* was first synthesized from diethyl malonate (10.00 g, 0.06 mol), 1-bromohexane (8.25 g, 0.05 mol) and sodium hydride (1.49 g, 0.06 mol) in DMF (abs., 50 mL) *(6)*. Colorless liquid; Yield: 7.42 g (73%); ^{1}H-NMR ($CDCl_3$, 500 MHz): δ / ppm = 4.20 (q, $^3J_{H,H}$ = 7.1 Hz, 4H, OCH_2), 3.30 (t, $^3J_{H,H}$ = 7.6 Hz, 1H, COCH), 1.87 (m, 2H, CHCH_2), 1.25 (m, 14H, CH_2), 0.88 (t, $^3J_{H,H}$ = 6.8 Hz, 3H, CH_3). The obtained diethyl 2-hexylmalonate (14.80 g, 0.06 mol) was alkylated with 1-bromotetradecane (33.83 g, 0.12 mol) and sodium hydride (2.93 g, 0.12 mol) in DMF (abs., 50 mL) *(6)*. Colorless solid; Yield: 26.19 g (97%); TLC (*n*-hexane): R_f = 0.10; ^{1}H-NMR ($CDCl_3$, 400 MHz): δ / ppm = 4.10 (q, $^3J_{H,H}$ = 7.1 Hz, 4H, OCH_2), 1.75 (m, 4H, CH(CH_2)$_2$), 1.16 (m, 38H, CH_2, OCH$_2$CH_3), 0.79 (m, 6H, CH_3).

Dimethyl 2-dodecyl-2-octylmalonate 3d. Synthesized from dimethyl 2-octylmalonate (10.00 g, 0.04 mol), 1-bromododecane (12.46 g, 0.05 mol) and sodium (1.91 g, 0.08 mol) in methanol (abs., 150 mL) *(7)*. Colorless liquid; Yield: 7.96 g (47%); TLC (*n*-hexane): R_f = 0.10; ^{1}H-NMR ($CDCl_3$, 400 MHz): δ / ppm = 3.70 (s, 6H, OCH_3), 1.86 (m, 4H, CH(CH_2)$_2$), 1.25 (m, 32H, CH_2), 0.88 (m, 6H, CH_3).

Dimethyl 2,2-didecylmalonate 3e. Synthesized from diethyl malonate (10.00 g, 0.06 mol), 1-bromodecane (28.75 g, 0.13 mol) and sodium (4.14 g, 0.18 mol) in methanol (abs., 150 mL) *(7)*. Colorless liquid; Yield: 9.90 g (40%); TLC (*n*-hexane): R_f = 0.10; ^{1}H-NMR ($CDCl_3$, 400 MHz): δ / ppm = 3.68 (s, 6H, OCH_3), 1.83 (m, 4H, CH(CH_2)$_2$), 1.23 (m, 32H, CH_2), 0.85 (t, $^3J_{H,H}$ = 6.8 Hz, 6H, CH_3).

General procedure for the synthesis of the branched carboxylates 4b-e *(9)*. A mixture of the alkyl substituted malonate (1 eq), LiCl (2 eq), and water (1 eq) in DMSO (150 mL) was stirred at reflux for 24 h. After cooling to room temperature water (150 mL) was added. The mixture was extracted with Diethylether (3 x 50 mL) and the combined organic layers washed with water (3 x 50 mL) After drying over anhydrous Na_2SO_4 the solvent was removed under reduced pressure. The residue was purified by column chromatography ($CHCl_3$ / *n*-hexane, 1 / 4 v/v).

Methyl 2-butyloctadecanoate 4b. Synthesized from dimethyl 2-butyl-2-hexadecylmalonate **3b** (25.24 g, 0.06 mol), water (1.09 g, 0.06 mol) and lithium chloride (5.17 g, 0.12 mol) in DMSO (abs., 50 mL). Colorless liquid; Yield: 10.46 g (48%); TLC (*n*-hexane/$CHCl_3$, 4/1 v/v): R_f = 0.90; ^{1}H-NMR ($CDCl_3$, 400 MHz): δ / ppm = 3.66 (s, 3H, OCH_3), 2.33 (m, 1H, C*H*), 1.56 (m, 2H, $CHCH_2$), 1.45 (m, 2H, $CHCH_2$), 1.25 (m, 36H, CH_2), 0.88 (m, 6H, CH_3).

Ethyl 2-hexylhexadecanoate 4c. Synthesized from diethyl 2-hexyl-2-tetradecylmalonate **3c** (26.19 g, 0.06 mol), water (1.06 g, 0.06 mol) and LiCl (7.52 g, 0.18 mol) in DMSO (abs., 50 mL). Colorless liquid; Yield: 19.60 g (90%); TLC (*n*-hexane/$CHCl_3$, 4/1 v/v): R_f = 0.90; ^{1}H-NMR ($CDCl_3$, 500 MHz): δ / ppm = 4.15 (q, $^3J_{H,H}$ = 7.1 Hz, 2H, OCH_2), 2.29 (m, 1H, C*H*), 1.57 (m, 2H, $CHCH_2$), 1.43 (m, 2H, $CHCH_2$), 1.25 (m, 39H, CH_2), 0.88 (m, 6H, CH_3).

Methyl 2-octyltetradecanoate 4d. Synthesized from dimethyl 2-dodecyl-2-octylmalonate **3d** (3.40 g, 8.32 mmol), water (0.15 g, 8.32 mmol) and lithium chloride (0.71 g, 16.64 mmol) in DMSO (abs., 50 mL). Colorless liquid; Yield: 1.70 g (58%); TLC (*n*-hexane/$CHCl_3$, 4/1 v/v): R_f = 0.90; ^{1}H-NMR ($CDCl_3$, 400 MHz): δ / ppm = 3.66 (s, 3H, OCH_3), 2.33 (m, 1H, C*H*), 1.59 (m, 2H, $CHCH_2$), 1.41 (m, 2H, $CHCH_2$), 1.25 (m, 36H, CH_2), 0.88 (m, 6H, CH_3).

Methyl-2-decyldodecanoate 4e. Synthesized from dimethyl 2,2-didecylmalonate **3e** (9.90 g, 0.02 mol), water (0.36 g, 0.02 mol) and LiCl (2.03 g, 0.05 mol) in DMSO (abs., 50 mL). Colorless liquid; Yield: 2.97 g (35%); TLC (*n*-hexane/$CHCl_3$, 4/1 v/v): R_f = 0.90; ^{1}H-NMR ($CDCl_3$, 400 MHz): δ / ppm = 3.66 (s, 3H, OCH_3), 2.33 (m, 1H, C*H*), 1.56 (m, 2H, $CHCH_2$), 1.45 (m, 2H, $CHCH_2$), 1.25 (m, 36H, CH_2), 0.88 (m, 6H, CH_3).

Procedure for the synthesis of the 2-alkylalkane-1-ols 5a-d *(10)*. The reaction was carried out under a argon atmosphere. $LiAlH_4$ (3 eq / ester group) was slowly suspended in dry diethyl ether (100 mL). The dialkylated malonate was dissolved in dry diethyl ether (100 mL) and added dropwise to this suspension. The mixture was heated to reflux for 6 h. After reaction water was added dropwise until the excess of $LiAlH_4$ was destroyed. The precipitate was dissolved by adding H_2SO_4 (10%, 50 mL) dropwise. The mixture was extracted with diethyl ether (3 x 50 mL) and the combined organic layers was washed with sat. aqu. $Na_2S_2O_3$, water and brine. After drying over anhydrous Na_2SO_4 the solvent was removed under reduced pressure and the residue purified by column chromatography ($CHCl_3$).

2-Butyloctadecane-1-ol 5b. Synthesized from methyl 2-butyloctadecanoate **4b** (23.30 g, 0.06 mol) and $LiAlH_4$ (10.00 g, 0.26 mol) in dry diethyl ether (250 mL). Colorless liquid; Yield: 15.67 g (73%); TLC ($CHCl_3$): R_f = 0.60; ^{1}H-NMR ($CDCl_3$, 400 MHz): δ / ppm = 3.54 (d, $^3J_{H,H}$ = 5.5 Hz, 2H, $HOCH_2$), 1.45 (m, 1H, CH_2CH), 1.25 (m, 36H, CH_2), 0.88 (m, 6H, CH_3).

2-Hexylhexadecane-1-ol 5c. Synthesized from Ethyl 2-hexylhexadecanoate **4c** (1.26 g, 3.41 mmol) and $LiAlH_4$ (0.39 g, 10.25 mmol) in dry diethyl ether (100 mL). Colorless liquid; Yield: 0.60 g (54%); TLC ($CHCl_3$): R_f = 0.60; ^{1}H-NMR ($CDCl_3$, 400 MHz): δ / ppm = 3.55 (t, $^3J_{H,H}$ = 4.9 Hz, 2H, $HOCH_2$), 1.45 (m, 1H, CH_2CH), 1.26 (m, 36H, CH_2), 1.14 (t, $^3J_{H,H}$ = 5.8 Hz, 1H, O*H*), 0.88 (t, $^3J_{H,H}$ = 6.8 Hz, 6H, CH_3).

2-Octyldodecane-1-ol 5d. Synthesized from methyl 2-octyltetradecanoate **4d** (1.76 g, 4.96 mmol) and $LiAlH_4$ (0.81 g, 21.51 mmol) in dry diethyl ether (100 mL). Colorless liquid; Yield: 1.05 g (65%); TLC ($CHCl_3$): R_f = 0.60; ^{1}H-NMR ($CDCl_3$, 400 MHz): δ / ppm = 3.55 (t, $^3J_{H,H}$ = 4.9 Hz, 2H, $HOCH_2$), 1.45 (m, 1H, CH_2CH), 1.26 (m, 36H, CH_2), 1.14 (t, $^3J_{H,H}$ = 5.8 Hz, 1H, O*H*), 0.88 (t, $^3J_{H,H}$ = 6.8 Hz, 6H, CH_3).

2-Decyldodecane-1-ol 5e. Synthesized from methyl 2-decyldodecanoate **4e** (2.97 g, 0.01 mol) and $LiAlH_4$ (1.14 g, 0.03 mol) in dry diethyl ether (100 mL). Colorless liquid; Yield: 2.09 g (80%); TLC ($CHCl_3$): R_f = 0.60; ^{1}H-NMR ($CDCl_3$, 400 MHz): δ / ppm = 3.55 (t, $^3J_{H,H}$ = 4.9 Hz, 2H, HOCH_2), 1.45 (m, 1H, CH_2C*H*), 1.26 (m, 36H, CH_2), 1.14 (t, $^3J_{H,H}$ = 5.8 Hz, 1H, O*H*), 0.88 (t, $^3J_{H,H}$ = 6.8 Hz, 6H, CH_3).

Procedure for the synthesis of the 2-alkyl-1-bromo-alkanes 2b-e *(11)***.** The appropriate alcohol **5** (1 eq), Bu_4NHSO_4 (5 mg) and conc. H_2SO_4 (2 mL) was suspended in HBr (48%, 50 mL) and heated to reflux for 24 h. After cooling to room temperature the mixture was extracted with diethyl ether (3 x 50 mL). The combined organic layers were washed with water and brine and dried over anhydrous Na_2SO_4. After removal of the solvent the residue was purified by column chromatography (*n*-hexane).

5-Bromomethylhenicosane 2b. Synthesized from 2-butyloctadecane-1-ol **5b** (3.39 g, 0.01 mol), HBr (48%, 50 mL), Bu_4NHSO_4 (0.05 g, 0.15 mmol) and H_2SO_4 (2 mL). Colorless liquid; Yield: 1.80 g (50%); TLC (*n*-hexane): R_f = 0.95; ^{1}H-NMR ($CDCl_3$, 400 MHz): δ / ppm = 3.45 (d, $^3J_{H,H}$ = 4.8 Hz, 2H, BrCH_2), 1.57 (m, 1H, C*H*), 1.26 (m, 36H, CH_2), 0.88 (m, 6H, CH_3).

7-Bromomethylhenicosane 2c. Synthesized from 2-hexylhexadecane-1-ol **5c** (600 mg, 1.84 mmol), HBr (48%, 50 mL), Bu_4NHSO_4 (0.05 g, 0.15 mmol) and H_2SO_4 (2 mL). Colorless liquid; Yield: 580 mg (81%); TLC (*n*-hexane): R_f = 0.95; ^{1}H-NMR ($CDCl_3$, 400 MHz): δ / ppm = 3.45 (d, $^3J_{H,H}$ = 4.8 Hz, 2H, BrCH_2), 1.57 (m, 1H, C*H*), 1.26 (m, 36H, CH_2), 0.88 (m, 6H, CH_3).

9-Bromomethylhenicosane 2d. Synthesized from 2-octyldodecane-1-ol **5d** (480 mg, 1.50 mmol), HBr (48%, 15mL), Bu_4NHSO_4 (0.05 g, 0.15 mmol) and H_2SO_4 (2 mL). Colorless liquid; Yield: 450 mg (77%); TLC (*n*-hexane): R_f = 0.95; ^{1}H-NMR ($CDCl_3$, 400 MHz): δ / ppm = 3.45 (d, $^3J_{H,H}$ = 4.7 Hz, 2H, BrCH_2), 1.57 (m, 1H, C*H*), 1.26 (m, 36H, CH_2), 0.88 (t, $^3J_{H,H}$ = 6.6 Hz, 6H, CH_3).

11-Bromomethylhenicosane 2e. Synthesized from 2-decyldodecane-1-ol **5e** (2.09 g, 6.40 mmol), HBr (48%, 50 mL), Bu_4NHSO_4 (0.05 g, 0.15 mmol) and H_2SO_4 (2 mL). Colorless liquid; Yield: 1.49 g (60%); TLC (*n*-hexane): R_f = 0.95; ^{1}H-NMR ($CDCl_3$, 400 MHz): δ / ppm = 3.45 (d, $^3J_{H,H}$ = 4.8 Hz, 2H, BrCH_2), 1.57 (m, 1H, C*H*), 1.26 (m, 36H, CH_2), 0.88 (m, 6H, CH_3).

Synthesis of the acetonides A*m/n*. A mixture of **1** and **2a-e**, K_2CO_3 (250 mg, 1.8 mmol) and Bu_4NI (5 mg) in anhydrous DMF (50 mL) was stirred at 80 °C for 12 h. After cooling to room temperature, the reaction was poured into water (50 mL) and the aqueous layer was extracted with Et_2O (3x50 mL). The combined organic layers were washed with saturated aqu. LiCl, water and brine. After drying over anhydrous Na_2SO_4, filtration and evaporation of the solvent, the crude product was purified by column chromatography (silica gel, $CHCl_3$/EtOAc, 4/1 v/v).

4,4''-Bis(1,2-isopropylidene-3-glyceryl)-2'-docosan-1-yloxy-*p*-terphenyl A20/0. Synthesized from **1** (400 mg, 0.8 mmol) and 1-bromodocosane (331 mg, 0.8 mmol). Purification by crystallisation from $CHCl_3$/PE. Colorless solid; Yield: 490 mg (75%). TLC ($CHCl_3$/EtOAc, 4/1 v/v): R_f = 0.95; ^{1}H-NMR ($CDCl_3$, *J*/Hz, 400 MHz): δ/ppm = 7.53(d, $^3J_{H,H}$ = 8.7 Hz, 2H, Ar-*H*), 7.49 (d, $^3J_{H,H}$ = 8.8 Hz, 2H, Ar-*H*), 7.32 (d, $^3J_{H,H}$ = 7.9 Hz, 1H, Ar-*H*),

7.16 (dd, $^3J_{H,H}$ = 8.0 Hz, $^4J_{H,H}$ = 1.5 Hz, 1H, Ar-*H*), 7.10 (s, 1H, Ar-*H*), 6.97 (d, $^3J_{H,H}$ = 8.7 Hz, 2H, Ar-*H*), 6.93 (d, $^3J_{H,H}$ = 8.7 Hz, 2H, Ar-*H*), 4.49 (m, 2H, OC*H*), 4.19 – 4.16 (m, 2H, OCH_2), 4.10 (dd, $^2J_{H,H}$ = 9.3 Hz, $^3J_{H,H}$ = 5.4 Hz, 2H, OCH_2), 4.01 – 3.95 (m, 4H, OCH_2), 3.92 (dd, $^2J_{H,H}$ = 8.5 Hz, $^3J_{H,H}$ = 5.6 Hz, 2H, OCH_2), 1.72 (tt, $^3J_{H,H}$ = 6.6 Hz, $^3J_{H,H}$ = 7.7 Hz, 2H, OCH_2CH_2), 1.46 (s, 6H, CH_3), 1.40 (s, 6H, CH_3), 1.40 (m, 2H, OCH_2CH_2CH_2), 1.39 – 1.24 (m, 36H, CH_2), 0.86 (t, $^3J_{H,H}$ = 6.4 Hz, 3H, CH_3).

4,4''-Bis(1,2-isopropylidene-3-glyceryl)-2'-(2-butyloctadec-1-yloxy)-*p*-terphenyl A16/4. Synthesized from **1** (200 mg, 0.4 mmol) and **2b** (170 mg, 0.4 mmol). Purification by column chromatography (silica gel, $CHCl_3$/EtOAc, 2/1 v/v). Colorless oil; Yield: 240 mg (75%). TLC ($CHCl_3$/EtOAc, 4/1 v/v): R_f = 0.95; ^{1}H-NMR ($CDCl_3$, *J*/Hz, 400 MHz): δ/ppm = 7.54 (d, $^3J_{H,H}$ = 8.4 Hz, 2H, Ar-*H*), 7.48 (d, $^3J_{H,H}$ = 8.5 Hz, 2H, Ar-*H*), 7.32 (d, $^3J_{H,H}$ = 7.9 Hz, 1H, Ar-*H*), 7.15 (d, $^3J_{H,H}$ = 7.9 Hz, 1H, Ar-*H*), 7.09 (s, 1H, Ar-*H*), 6.97 (d, $^3J_{H,H}$ = 8.5 Hz, 2H, Ar-*H*), 6.92 (d, $^3J_{H,H}$ = 8.5 Hz, 2H, Ar-*H*), 4.47 (quin., $^3J_{H,H}$ = 5.9 Hz, 2H, OC*H*), 4.17 (m, 2H, OCH_2), 4.09 (m, 2H, OCH_2), 3.99 – 3.89 (m, 4H, OCH_2), 3.87 (d, $^3J_{H,H}$ = 5.4 Hz, 2H, OCH_2CH) 1.70 (m, 1H, OCH_2C*H*), 1.47 (s, 6H, C(CH_3)$_2$), 1.40 (s, 6H, C(CH_3)$_2$), 1.23 (m, 36H, CH_2), 0.85 (m, 6H, CH_3).

4,4''-Bis(1,2-isopropylidene-3-glyceryl)-2'-(2-hexylhexadec-1-yloxy)-*p*-terphenyl A14/6. Synthesized from **1** (200 mg, 0.4 mmol) and **2c** (170 mg, 0.4 mmol). Purification by column chromatography (silica gel, $CHCl_3$/EtOAc, 4/1 v/v). Colorless oil; Yield: 280 mg (87%). TLC ($CHCl_3$/EtOAc, 4/1 v/v): R_f = 0.95; ^{1}H-NMR ($CDCl_3$, *J*/Hz, 400 MHz): δ/ppm = 7.55 (d, $^3J_{H,H}$ = 8.7 Hz, 2H, Ar-*H*), 7.50 (d, $^3J_{H,H}$ = 8.7 Hz, 2H, Ar-*H*), 7.34 (d, $^3J_{H,H}$ = 7.8 Hz, 1H, Ar-H), 7.17 (dd, $^3J_{H,H}$ = 7.8 Hz, $^4J_{H,H}$ = 1.5 Hz, 1H, Ar-*H*), 7.11 (s, 1H, Ar-*H*), 7.00 (d, $^3J_{H,H}$ = 8.7 Hz, 2H, Ar-*H*), 6.94 (d, $^3J_{H,H}$ = 8.7 Hz, 2H, Ar-*H*), 4.50 (m, 2H, OC*H*), 4.20 (m, 2H, OCH_2), 4.10 (m, 2H, OCH_2), 4.00 (m, 2H, OCH_2), 3.94 (m, 2H, OCH_2), 3.89 (d, $^3J_{H,H}$ = 5.4 Hz, 2H, OCH_2CH), 1.72 (m, 1H, OCH_2C*H*), 1.48 (s, 6H, OC(CH_3)$_2$), 1.42 (s, 6H, OC(CH_3)$_2$), 1.38 – 1.17 (m, 36H, CH_2), 0.88 (m, 6H, CH_3).

4,4''-Bis(1,2-isopropylidene-3-glyceryl)-2'-(2-octyltetradec-1-yloxy)-*p*-terphenyl A12/8. Synthesized from of **1** (200 mg, 0.4 mmol) and **2d** (170 mg, 0.4 mmol). Purification by column chromatography (silica gel, $CHCl_3$/MeOH, 9/1 v/v). Colorless oil; Yield: 250 mg (78%). TLC ($CHCl_3$/EtOAc, 4/1 v/v): R_f = 0.95; ^{1}H-NMR ($CDCl_3$, *J*/Hz, 500 MHz): δ/ppm = 7.54 (d, $^3J_{H,H}$ = 8.8 Hz, 2H, Ar-*H*), 7.50 (d, $^3J_{H,H}$ = 8.8 Hz, 2H, Ar-*H*), 7.34 (d, $^3J_{H,H}$ = 7.8 Hz, 1H, Ar-*H*), 7.17 (dd, $^3J_{H,H}$ = 7.8 Hz, $^4J_{H,H}$ = 1.8 Hz, 1H, Ar-*H*), 7.11 (d, $^4J_{H,H}$ = 1.6 Hz, 1H, Ar-*H*), 6.99 (d, $^3J_{H,H}$ = 8.8 Hz, 2H, Ar-*H*), 6.94 (d, $^3J_{H,H}$ = 8.8 Hz, 2H, Ar-*H*), 4.51 (quin., $^3J_{H,H}$ = 5.9 Hz, 2H, OC*H*), 4.20 (m, 2H, OCH_2), 4.11 (m, 2H, OCH_2), 3.98 – 3.93 (m, 4H, OCH_2), 3.89 (d, $^3J_{H,H}$ = 5.4 Hz, 2H, OCH_2CH) 1.79 (m, 1H, OCH_2C*H*), 1.48 (s, 6H, C(CH_3)$_2$), 1.42 (s, 6H, C(CH_3)$_2$), 1.25 (m, 36H, CH_2), 0.84 (t, $^3J_{H,H}$ = 6.8 Hz, 6H, CH_3).

4,4''-Bis(1,2-isopropylidene-3-glyceryl)-2'-(2-decyldodec-1-yloxy)-*p*-terphenyl A10/10. Synthesized from **1** (300 mg, 0.6 mmol) and **2**e (250 mg, 0.6 mmol), light brownish solid; Yield: 350 mg (86%); mp. 57 °C. TLC ($CHCl_3$/EtOAc, 4/1 v/v): R_f = 0.95; ^{1}H-NMR ($CDCl_3$, *J*/Hz, 400 MHz): δ/ppm = 7.53 (d, $^3J_{H,H}$ = 8.7, 2H, Ar-*H*), 7.48 (d, $^3J_{H,H}$ = 8.7, 2H, Ar-*H*), 7.32 (d, $^3J_{H,H}$ = 7.9, 1H, Ar-*H*), 7.15 (dd, $^3J_{H,H}$ = 7.7, $^4J_{H,H}$ = 1.5, 1H, Ar-*H*), 7.10 (d, $^4J_{H,H}$ = 1.5, 1H, Ar-*H*), 6.98 (d, $^3J_{H,H}$ = 8.7, 2H, Ar-*H*), 6.92 (d, $^3J_{H,H}$ = 8.9, 2H, Ar-*H*), 4.49 (quin., $^3J_{H,H}$ = 5.8, 2H, OC*H*), 4.17 (m, 2H, OCH_2), 4.12-4.08 (m, 2H, OCH_2), 3.99-3.96 (m, 2H, OCH_2), 3.93-3.91 (m, 2H, OCH_2), 3.90-3.87 (m, 2H, OCH_2CH), 1.70 (m, 1H, OCH_2C*H*), 1.47 (s, 6H, C(CH_3)$_2$), 1.40 (s, 6H, C(CH_3)$_2$), 1.25-1.23 (m, 36H, CH_2), 0.86 (t, $^3J_{H,H}$ = 7.1, 6H, CH_3).

Synthesis and analytical data of compounds *n/m*. Deprotection with pyridinium p-toluenesulfonate (PPTS) *(12)* was used for **20/0-16/4** and **10/10**. A mixture of **A*m/n*** and PPTS (170 mg, 0.70 mmol) in MeOH (50 mL) was stirred at 50 °C for 1 d. The progress of the reaction was monitored via TLC. The solvent was evaporated and the residue dissolved in water and CH_2Cl_2. The phases were seperated and the water phase extracted three times with CH_2Cl_2 (3x25 mL). The organic layer was washed with sat. aq. $NaHCO_3$ (25 mL) and brine (25 mL). After drying over anhydrous Na_2SO_4, filtration and evaporation of the solvent to compound was purified as detailed below.

3,3'-[2´-(Docos-1-yloxy)- 4,4''-*p*-terphenylen]diglyerol 20/0. Synthesized from **A20/0** (490 mg, 0.60 mmol). Purification by crystallization MeOH/n-pentane. Colorless solid; Yield: 370 mg (85%); mp = 34 °C; TLC ($CHCl_3$/MeOH, 4/1 v/v): R_f = 0.70; ^{1}H-NMR (pyridine-d$_5$, *J*/Hz, 400 MHz): δ/ppm = 7.79 (m, 3H, Ar-*H*), 7.56 (m, 2H, Ar-*H*), 7.52 (m, 2H, Ar-*H*), 7.20 (m, 4H, Ar-*H*), 5.86 (mb, 4H, O*H*), 4.64 – 4.58 (m, 6H, OC*H*, OC*H*$_2$), 4.57 – 4.55 (m, 2H, OC*H*$_2$), 4.24 (m, 2H, OC*H*$_2$), 4.01 (d, $^3J_{H,H}$ = 6.3 Hz, 2H, OC*H*$_2$), 1.77 (tt, $^3J_{H,H}$ = 7.1 Hz, $^3J_{H,H}$ = 6.2 Hz, 2H, OCH_2CH_2), 1.44 (m, 2H, $OCH_2CH_2CH_2$), 1.30 – 1.24 (m, 36H, C*H*$_2$), 0.85 (t, $^3J_{H,H}$ = 6.7 Hz, 3H, C*H*$_3$). ^{13}C-NMR (pyridine-d$_5$, 125 MHz): δ/ppm = 14.3, 22.9, 26.4, 29.6, 29.6, 29.9, 30.0, 32.1 (*C*H$_2$, *C*H$_3$), 64.4, 64.4, 68.7, 71.0, 71.1, 71.5 (O*C*H, O*C*H$_2$), 111.5, 114.7, 115.6, 119.6, 123.0, 128.5. 128.9, 129.3, 131.3, 141.3, 156.9, 159.0, 159.6 (Ar-*C*). HRMS (m/z): $[M]^+Cl^-$ calcd. for $C_{46}H_{70}O_7Cl$, 769.4805; found, 769.4816; analysis (calcd. for $C_{46}H_{70}O_7$): C (75.16, 75.45), H (9.60, 9.58).

3,3'-[2´-(2-Butyloctadec-1-yloxy) 4,4''-*p*-terphenylen]diglyerol 16/4. Synthesized from **A16/4** (240 mg, 0.29 mmol). Purification by column chromatography (silica gel, THF/EtOAc, 1/1 v/v) and crystallization from THF/*n*-Hexan. Colorless solid; Yield: 170 mg (80%); mp = 43 °C; TLC ($CHCl_3$/MeOH, 4/1 v/v): R_f = 0.70; ^{1}H-NMR (Pyridin-d$_5$, J/Hz, 500 MHz): δ/ppm = 7.83 (m, 2H, Ar-*H*), 7.78 (m, 2H, Ar-*H*), 7.55 (m, 2H, Ar-*H*), 7.43 (dd, $^3J_{H,H}$ = 7.8 Hz, $^4J_{H,H}$ = 1.6 Hz, 1H, Ar-*H*), 7.27 (m, 4H, Ar-*H*), 6.92 (d, $^3J_{H,H}$ = 18.9 Hz, 2H, O*H*), 6.51 (d, $^3J_{H,H}$ = 23.4 Hz, 2H, O*H*), 4.66 – 4.56 (m, 4H, OC*H*$_2$), 4.51 (m, 2H, CH_2CHCH_2), 4.27 (m, 4H, OC*H*$_2$), 4.08 (d, $^3J_{H,H}$ = 5.3 Hz, 2H, OC*H*$_2$CH), 1.82 (m, 1H, OCH_2CH), 1.53 (m, 2H, C*H*$_2$), 1.47 – 1.19 (m, 34H, C*H*$_2$), 0.88 (t, $^3J_{H,H}$ = 6.8 Hz, 6H, C*H*$_3$). ^{13}C-NMR (Pyridin-d$_5$, 100 MHz): δ /ppm = 14.2, 14.3, 22.9, 23.3, 27.2, 29.3, 29.6, 29.9, 30.0, 30.0, 30.4, 31.5, 31.9, 32.1, 38.4 (*C*H$_2$, *C*H$_3$), 64.4, 64.4 (O*C*H), 69.5, 69.7, 69.8, 70.1, 70.1 (O*C*H$_2$), 109.9, 113.2, 114.1, 118.1, 121.5, 127.1, 128.0, 129.8, 132.4, 155.8, 157.5, 158.2 (Ar-*C*). HRMS (m/z): $[M]^+Cl^-$ calcd. for $C_{46}H_{70}O_7Cl$, 769.4805; found, 769.4811; analysis (calcd. for $C_{46}H_{70}O_7$): C (75.16, 75.05), H (9.60, 9.67).

3,3'-[2´-(2-Hexylhexadec-1-yloxy)- 4,4''-*p*-terphenylen]diglyerol 14/6. Synthesized from **A14/6** (280 mg, 0.34 mmol). Purification by column chromatography (silica gel, $CHCl_3$/MeOH, 4/1 v/v) and crystallization from MeOH. Colorless solid; Yield: 210 mg (83%); mp = 49 °C; TLC ($CHCl_3$/MeOH, 4/1 v/v): R_f = 0.70; ^{1}H-NMR (Pyridin-d$_5$, J/Hz, 500 MHz): δ/ppm = 7.86 – 7.77 (m, 4H, Ar-*H*), 7.58 – 7.53 (m, 2H, Ar-*H*), 7.43 (dd, $^3J_{H,H}$ = 7.9 Hz, $^3J_{H,H}$ = 1.6 Hz, 1H, Ar-*H*), 7.32 – 7.24 (m, 4H, Ar-*H*), 6.91 (d, $^3J_{H,H}$ = 19.8 Hz, 1H, O*H*), 6.51 (d, $^3J_{H,H}$ = 23.7 Hz, 1H, O*H*), 4.60 (m, 4H, OC*H*$_2$), 4.51 (m, 2H, CH_2CHCH_2), 4.27 (m, 4H, OC*H*$_2$), 4.10 (d, $^3J_{H,H}$ = 5.3 Hz, 2H, OC*H*$_2$CH), 1.85 (m, 1H, OCH_2CH), 1.55 (m, 2H, C*H*$_2$), 1.49 – 1.42 (m, 2H, C*H*$_2$), 1.42 – 1.18 (m, 32H, C*H*$_2$), 0.88 (dt, $^3J_{H,H}$ = 7.1 Hz, $^3J_{H,H}$ = 3.4 Hz, 6H, C*H*$_3$). ^{13}C-NMR (Pyridin-d$_5$, 100 MHz): δ /ppm = 14.0, 14.0, 22.7, 22.7, 26.9, 26.9, 29.3, 29.7, 29.7, 29.7, 29.8, 29.8, 30.1, 31.6, 31.8, 31.9, 38.2 (*C*H$_2$, *C*H$_3$), 64.1, 64.2 (O*C*H), 70.7, 70.8, 71.0, 71.3, 71.3 (O*C*H$_2$), 111.1, 114.4, 115.3, 119.3, 128.3, 129.2, 131.0,

131.1, 133.6, 141.2, 156.9, 158.7, 159.4 (Ar-*C*). HRMS (m/z): $[M]^+Cl^-$ calcd. for $C_{46}H_{70}O_7Cl$, 769.4805; found, 769.4796; analysis (calcd. for $C_{46}H_{70}O_7$): C (75.16, 75.27), H (9.60, 9.80).

3,3’-[2´-(2-Octyltetradec-1-yloxy)- 4,4’’-*p*-terphenylen]diglyerol 12/8. Deprotection with HCl *(13)*. A mixture of **A12/8** (250 mg, 0.31 mmol) and 10% HCl (5 mL) in MeOH (20 mL) was heated to reflux for 5 h. The progress of the reaction was monitored by TLC. The solvent was evaporated and the residue was dissolved in EtOAc, washed with sat. aq. $NaHCO_3$ (25 mL), water (25 mL) and brine (25 mL). After drying over anhydrous Na_2SO_4, filtration and evaportation of the solvent, the crude product was purified by column chromatography (silica gel, $CHCl_3$/MeOH, 9/1 v/v) and crystallization from MeOH. Colorless solid; Yield: 150 mg (66%); mp. 72 °C; TLC ($CHCl_3$/MeOH, 4/1 v/v): R_f = 0.70; ^{1}H-NMR (Pyridin-d$_5$, *J*/Hz, 400 MHz): δ/ppm = 7.81 (dd, $^3J_{H,H}$ = 8.7 Hz, $^4J_{H,H}$ = 2.1 Hz, 4H, Ar-*H*), 7.57 – 7.53 (m, 2H, Ar-*H*), 7.43 (dd, $^3J_{H,H}$ = 7.8 Hz, $^3J_{H,H}$ = 1.5 Hz, 1H, Ar-*H*), 7.33 – 7.24 (m, 4H, Ar-*H*), 4.60 (m, 4H, OC*H*$_2$), 4.55 – 4.47 (m, 2H, CH$_2$C*H*CH$_2$), 4.32 – 4.21 (m, 4H, OC*H*$_2$), 4.12 (d, $^3J_{H,H}$ = 5.2 Hz, 2H, OC*H*$_2$CH), 1.94 – 1.80 (m, 1H, OCH$_2$C*H*), 1.62 – 1.19 (m, 36H, C*H*$_2$), 0.88 (dd, $^3J_{H,H}$ = 6.8, $^3J_{H,H}$ = 6.0 Hz, 6H, C*H*$_3$). ^{13}C-NMR (Pyridin-d$_5$, 100 MHz): δ /ppm = 12.9, 21.5, 25.7 28.2, 28.4, 28.5, 28.5, 28.6, 28.6, 28.9, 28.9, 30.5, 30.7, 37.1 (*C*H$_2$, *C*H$_3$), 62.9, 63.0 (O*C*H), 69.5, 69.7, 69.8, 70.1, 70.1 (O*C*H$_2$), 109.9, 113.2, 114.1, 118.1, 121.5, 127.1, 128.0, 129.8, 132.4, 155.8, 157.5, 158.2 (Ar-*C*). HRMS (m/z): $[M]^+Cl^-$ calcd. for $C_{46}H_{70}O_7Cl$, 769.4805; found, 769.4802; analysis (calcd. for $C_{46}H_{70}O_7$): C (75.16, 75.38), H (9.60, 9.54).

3,3’-[2´-(2-Decyldodec-1-yloxy)- 4,4’’-*p*-terphenylen]diglyerol 10/10. Synthesized from **A10/10** (350 mg, 0.50 mmol). Purified by crystallization from MeOH/*n*-pentane. Colorless solid; Yield: 120 mg (35%); mp = 75 °C; TLC ($CHCl_3$/MeOH, 4/1 v/v): R_f = 0.70; ^{1}H-NMR (DMSO-d$_6$, *J*/Hz, 500 MHz): δ/ppm = 7.65 (d, $^3J_{H,H}$ = 8.6, 2H, Ar-*H*), 7.44 (d, $^3J_{H,H}$ = 8.7, 2H, Ar-*H*), 7.30 (d, $^3J_{H,H}$ = 7.7, 1H, Ar-*H*), 7.24 (s, 1H, Ar-*H*), 7.22 (d, $^3J_{H,H}$ = 7.8, 1H, Ar-*H*), 7.02 (d, $^3J_{H,H}$ = 8.7, 2H, Ar-*H*), 6.93 (d, $^3J_{H,H}$ = 8.8, 2H, Ar-*H*), 4.97-4.93 (m, 2H, O*H*), 4.68-4.64 (m, 2H, O*H*), 4.10-4.00 (m, 2H, OC*H*$_2$), 3.94 (m, 2H, OC*H*$_2$), 3.92-3.86 (m, 2H, OCH$_2$C*H*), 3.81 (m, 2H, OC*H*$_2$), 3.46 (m, 4H, OC*H*$_2$), 1.66 (m, 1H, OCH$_2$C*H*), 1.33-1.21 (m, 36H, C*H*$_2$), 0.83 (t, $^3J_{H,H}$ = 6.7, 6H, C*H*$_3$). ^{13}C-NMR (DMSO-d$_6$, 125 MHz): δ/ppm = 14.4, 22.5, 26.5, 29.1, 29.3, 29.4, 29.7, 31.2, 31.7, 37.7, 39.5, 39.6, 39.8, 39.9, 40.0, 40.1, 40.2, 40.3, 40.4, 40.5, 40.6 (*C*H$_2$, *C*H$_3$), 63.2, 63.3 (O*C*H), 70.1, 70.2, 70.4, 70.5, 79.6 (O*C*H$_2$), 114.2, 115.3, 118.9, 128.2, 128.5, 130.5, 130.7, 130.9, 132.7, 140.4, 156.5, 158.2, 159.0 (Ar-*C*). HRMS (m/z): $[M]^+Cl^-$ calcd. for $C_{46}H_{70}O_7Cl$, 769.4805; found, 769.4815; analysis (calcd. for $C_{46}H_{70}O_7 \cdot H_2O$): C (73.31, 73.51), H (9.64, 9.51

Supplementary References

1 Miyaura, N., Yanagi, T., Suzuki, A. The Palladium-Catalyzed Cross-Coupling Reaction of Phenylboronic Acid with Haloarenes in the Presence of Bases. *Synth. Commun.* **11**, 513-519 (1981).

2 Henley, R. V., Turner, E. E. The scission of diaryl ethers and related compounds by means of piperidine. Part III. The nitration of 2:4-dibromo-2’:4’-dinitrophenyl ether and of 2:4-dibromophenyl *p*-toluenesulphonate and benzoate. The chlorination and bromination of *m*-nitrophenol. *J. Chem. Soc.*, 928-940 (1930).

3 Liu, H., Bernhardsen, M., Fiksdahl, A. Polybrominated diphenyl ethers (BDEs); preparation of reference standards and fluorinated internal analytical standards. *Tetrahedron* **62**, 3564-3572 (2006).

4 Kölbel, M., Beyersdorff, T., Tschierske, C., Diele, S., Kain, J. Thermotropic and Lyotropic Liquid Crystalline Phases of Rigid Aromatic Amphiphiles. *Chem. Eur. J.* **6**, 3821-3837 (2000).

5 Kölbel, M., Beyersdorff, T., Cheng, X., Tschierske, C., Kain, J., Diele, S. Design of Liquid Crystalline Block Molecules with Nonconventional Mesophase Morphologies: Calamitic Bolamaphiphiles with Lateral Alkyl Chains. *J. Am. Chem. Soc.* **123**, 6809-6818 (2001).
6 Pokholenko, O., Gissot, A., Vialet, B., Bathany, K., Thiéry, A., Barthélémy, P. Lipid oligonucleotide conjugates as responsive nanomaterials for drug delivery. *J. Mater. Chem. B* **1**, 5329-5334 (2013).
7 Prehm, M., Liu, F., Zeng, X., Ungar, G., Tschierske, C. Axial-Bundle Phases – New Modes of 2D, 3D and Helical Columnar Self-Assembly in Liquid Crystalline Phases of Bolaamphiphiles with Swallow Tail Lateral Chains. *J. Am. Chem. Soc.* **133**, 4906-4916 (2011).
8 Dox, A. W. Ethyl-Normal-Hexylbarbituric Acid and other Derivatives of Normal-Hexylmalonic Acid. *J. Am Chem. Soc.* **46**, 1707-1711 (1924).
9 Krapcho, A. P., Weimaster, J. F., Eldridge, J. M., Jahngen Jr, E. G. E., Loves, A. J., Stephens, W. P. Synthetic applications and mechanism studies of the decarbalkoxylations of germinal diesters and related systems effected in dimethyl sulfoxide by water and/or by water with added salts. *J. Org. Chem.* **43**, 138-147 (1978).
10 Nystrom, R. F., Brown, W. G. Reduction of Organic Compounds by Lithium Aluminium Hydride. I. Aldehydes, Ketones, Esters, Acid Chlorides and Acid Anhydrides. *J. Am. Chem. Soc.* **69**, 1197-1199 (1947).
11 Dakka, G., Sasson, Y. Selective hydrobromination of branched alcohols using phase transfer catalysis. *Tetrahedron Lett.* **28**, 1223-1224 (1987).
12 van Rijsbergen, R., Anteunis, M. J. O., De Bruyn, A. Selective Removal of the Isopropylidene Group in 4-O-Protected 1,6-Anhydro-2,3-O-Isopropyledene-β-D-Mannopyranose and the Conformational Impact of it. *J. Carbohydr. Chem.* **2**, 395-404 (1983).
13 Angyal, S. J., Beveridge, R. J. Intramolecular acetal formation by primary *versus* secondary hydroxyl groups. *Carbohydr. Res.* **65**, 229-234 (1978).
14 Immirzi, A., Perini, B. Prediction of density in organic crystals. *Acta Cryst.* A **33**, 216-218 (1977).
